# Thin-Layer Sample–Ion-Selective Nanomembrane Architecture for Coulometric Cation Sensing

**DOI:** 10.1021/acs.analchem.6c01562

**Published:** 2026-09-22

**Authors:** Nuria Martínez-Lorca, Yujie Liu, Gastón A. Crespo, María Cuartero

**Affiliations:** † UCAM-SENS, Universidad Católica San Antonio de Murcia, UCAM HiTech, Avda. Andrés Hernández Ros, 1, 30107 Murcia, Spain; ‡ Department of Chemistry, School of Engineering Science in Chemistry, Biotechnology and Health, KTH Royal Institute of Technology, Teknikrigen 30, SE-100 44 Stockholm, Sweden

## Abstract

Herein, we investigate a coulometric nanomembrane-based ISE integrated into a microfluidic cell pursuing the absolute analysis of potassium ions (K^+^), selected as a model example. The sensor consisted of a glass substrate coated with the conducting polymer poly­(3-octylthiophene) (POT) and a nanometer-sized, potassium-selective membrane casted on top. In the microfluidic cell, the sample is confined to a thin-layer domain being sandwiched between the working electrode and the counter/reference electrode. Two electrochemical protocols are developed toward exhaustive ion transfer at the membrane-sample interface: (i) an anodic mode, where K^+^ is first accumulated in the membrane and then released to the sample, and (ii) a cathodic mode, where ion release from the membrane is followed by a complete uptake. Tuned POT oxidation and reduction are respectively utilized as the driving force for the ultimate ion transfer to be analytically exploited, with linear sweep voltammetry (LSV) and chronoamperometry being explored as charge readout strategies. The calibration-free operation is validated for the anodic LSV protocol within a range of 1–30 μM; while other routes exhibited deviations from the 100% coulometric efficiency that may arise from several factors: (i) variations in the sample volume due to the spacer material, (ii) lateral diffusion in the microfluidic channel, and (iii) interferences. A fundamental asymmetry was observed between ion uptake and release at the membrane interface, meaning that calibration-free operation via Faradaic quantification relies heavily on the specific electrochemical protocol applied. The developed sensor was tested for the quantification of K^+^ in diverse matrices, including urine, blood serum, environmental water, and standard solutions, showing overall good agreement with ion chromatography when appropriate protocols were employed.

## Graphical abstract

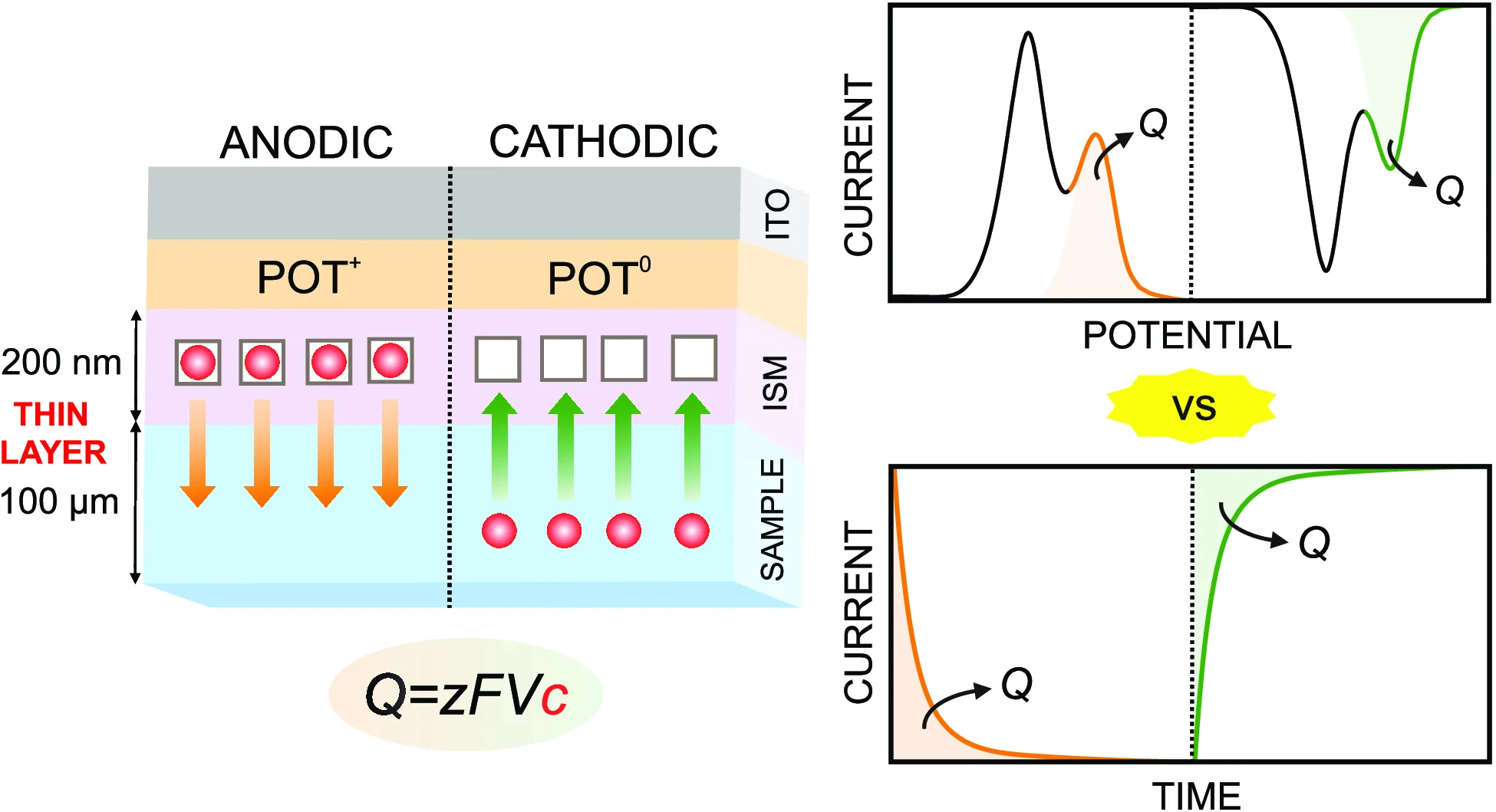

Existing demands for portable and autonomous analytical instruments across several domains have amplified research initiatives focused on the development of electrochemical sensors that can deliver direct and reliable quantification of ionic species. Ion-selective electrodes (ISEs) are particularly pertinent because of their simplicity, cost-effectiveness, and adaptability to various sample types.^1^ Conventional ISEs necessitate regular calibration to address response variations generated from different factors, which limits their long-term utility and field application.^2,3^ Effectively, the implementation of calibration-free ISEs is expected to enhance their precision and broaden their range of applications. The main goal is to obtain the analyte concentration directly from the raw signal, and coulometry possesses this potential. It is a charge-based technique that calculates concentration through the Faraday’s law, providing absolute observations when several strict conditions are met. These include that the sample volume must be well-defined, the analyte must undergo complete transformation and/or transport, and the electrochemical efficiency must approach 100%, even if there are several interconnected subprocesses or reactions.^4^ Furthermore, to ensure a practical time scale (seconds-minutes), thin-layer configurations for the elements integrating the coulometric sensor are required.

Pioneering works about the demonstration of coulometric detection of ions were performed at the interface between two immiscible liquids. It was shown that complete electrolysis is achievable under controlled geometries.^5,6^ Overall, the corresponding studies established the theoretical foundation for absolute charge-to-concentration conversion from various electrochemical readouts of ions. Later developments explored ion-selective membranes (ISMs) to pursue selectivity in the ion transfer process, therefore extending coulometric detection to sensor formats closer to those needed for real applications.^5,7–13^ For example, polymeric or polypropylene-based membranes doped with ionophores were described to quantify cations (K^+^,^5,7,8^ Ca^2+^ ^10^) and anions (NO_2_^–^ ^11^ and NO_3_^–^ ^12^). In essence, a constant potential is applied to selectively induce ion transport across the membrane–sample interface. The integration of the resulting current yields the associated charge from which the ion concentration in the sample is calculated. Notably, the investigation of the device architecture is key toward improved coulometric readouts. In that sense, tubular sensors facilitate confined sample geometries,^5,7,10,12^ whereas flat and microfluidic configurations allow a more precise control of the sample volume and faster mass transport.^8,9,11^ Also, paper-based or laminated thin-film cells have been proposed.^13^

The configuration of ISEs has been claimed to play a crucial role in shaping the coulometric performance. Reducing the thickness of the ISM has been an effective strategy to accelerate mass transport and improve response sharpness, which has been specifically demonstrated for voltammetric ISEs.^9,14–16^ Spin-coated films, often below 10 μm of thickness, have exhibited faster equilibration and lower resistance compared to thicker, drop-casted membranes. When the membrane thickness was further decreased, reaching hundreds of nanometers (e.g., ca. 300 nm), mass transport in the ISM has demonstrated to be totally neglectable.^9^ However, it is important to note that the membrane thickness is not the only parameter governing mass transport within the ISM. The equilibration time scale also depends on the diffusion coefficients of the species involved, which are strongly influenced by the membrane composition. For instance, previous studies employing nonpolymeric or liquid–liquid systems have demonstrated rapid ion transport using these thicker interfaces due to substantially higher diffusion coefficients.^17,18^ In contrast, in polymeric membranes, where diffusion is typically orders of magnitude slower, reducing the ISM thickness to the nanoscale becomes an effective strategy to ensure rapid response times.

A promising direction is to have these membranes in tandem with a thin-layer sample. Under such dual confinement, mass transport in the entire sensor device is no longer limited by diffusion, which is in principle ideal toward a complete (or absolute) analyte transport and/or conversion within a short practical time. Then, correcting nonfaradaic contributions in the read current (e.g., by potential-pulse compensation or geometric optimization),^11,19^ the corresponding charge represents the exact amount of ion analyte in the sample. While some progress has been made in integrating coulometric transduction with solid-state ISEs facing thin-layer samples,^8,19^ the development of robust devices that ensure reproducible performance remains an active area of research, specially concerning the additional use of thin membrane-based ISEs. Existing systems often demonstrate significant deviations of the theoretical behavior (>20%), rely on empirical corrections to ensure accuracy but that can somehow “force” the data in such a direction, and are rarely applied to real samples, in contrast to the present work.

The development of sensors capable of directly measuring the analyte concentration in a sample without any prior calibration is a challenge in electroanalysis. To address it, herein, we have developed a coulometric strategy based on thin-layer samples (<100 μm in thickness) combined to nanometer-sized ISMs (ca. 300 nm in thickness), investigating the complete analyte depletion through the direct correspondence of the charge readout with the cation analyte concentration. The sensing platform integrates a conducting polymer poly­(3-octylthiophene) (POT) with a K^+^-selective ultrathin membrane on top, establishing a coupling between the electron-transfer at the electrode-POT interface and selective ion-transfer at the membrane-sample interface. Based on this charge transfer mechanism, the membrane can operate as either donor or receptor of ions upon activation through anodic or cathodic sweep or chrono- potential. In the anodic mode, cations are first accumulated in the membrane and subsequently expelled during POT oxidation, whereas the cathodic mode involves the initial depletion of any cation present in the membrane followed by the selective uptake of the cation analyte (potassium ion in this case, K^+^) from the sample into the membrane. The resulting electrochemical signals are integrated to quantify the charge, which correlates with the K^+^ concentration in the sample via the Faraday Law.

While previous studies established the foundations for the analytical exploitation of the voltammetric response of ISEs based on nanometer-sized ISMs,^20^ coulometric interrogation remains unexplored. Effectively, peak potentials and currents have traditionally been evaluated as the primary quantitative signals; however, the potential of charge has only been briefly mentioned, and pulse techniques have never been introduced to interrogate the system, to the best of our knowledge.^9^ An important aspect is the understanding of any possible asymmetry between anodic and cathodic paths (i.e., release versus accumulation of the main cation), which is here addressed through a systematic comparison of four interrogation protocols considering LSV and chronoamperometry. Establishing a protocol and methodology toward a calibration-free approach is far from trivial, being thoroughly investigated in this work and successfully translated into a validated real sample analysis. Notably, the key factors governing the coulometric efficiency of faradaic concentration quantification are discussed, providing a practical guideline for the further development of this concept toward a truly calibration-free analytical option.

## Experimental Section

### Reagents Materials and Equipment

Polyurethane (PU, Selectophore), bis­(2-ethylhexyl)­sebacate (DOS), sodium tetrakis­[3,5-bis­(trifluoromethyl)­phenyl]­borate (NaTFPB), potassium ionophore I (valinomycin), 3-octylthiophene (97%, OT), tetrahydrofuran (anhydrous, ≥99.9%, THF) and high purity grade sodium chloride Selectophore were purchased from Sigma-Aldrich. Lithium perchlorate (anhydrous, 99%, LiClO_4_), acetonitrile (anhydrous, ≥99.5%, ACN), absolute ethanol (≥99.5%), potassium chloride standard solution (0.1 M) and potassium chloride (99.5%) were acquired in VWR. Aqueous solutions were prepared by dissolving the appropriate salts in deionized water (>18.2 MΩ.cm). Indium tin oxide (ITO) coated glass slides (10 mm × 35 mm × 1.1 mm, transmittance >83%, surface resistivity <10 Ω/sq) were sourced from Zhuhai Kaivo Optoelectronic Technology. The PTFE and the copper tape were purchased from RS Components. The silicon rubber was supplied by Junying CNClathing company.

LSV and chronoamperometry experiments were performed using a VIONIC potentiostat controlled with INTELLO 1.5 software (supplied by Metrohm). To ensure accurate signal acquisition and minimize instrumental noise, the electrochemical cell was placed inside a Faraday cage (purchased in Rittal). Measurements were conducted using a fixed current range (typically 10 μA) to prevent registration delays or artifacts associated with automatic ranging. Additionally, a sampling rate of 100 ms was employed to provide sufficient temporal resolution for precise peak integration. A High Precision Multichannel Pump model ISM935c from ISMATEC was used for the sample injection. A Metrohm 940 Professional Ion Chromatography (IC) instrument equipped with a Metrosep C6–150/4.0 column and 889 IC Sample Center as Autosampler (sample eluent of 4 mM HNO_3_, injection volume of 20 μL, flow rate of 0.9 mL/min) was employed to validate the real samples analysis. Calculations were accomplished in MATLAB_R2022b software.

The K^+^ content was evaluated in various real samples, including blood equine serum, urine, canal water and a standard solution. Prior to analysis, all samples were stored under refrigeration and filtered using 0.45 μm-pore size filters coupled to syringes to remove suspended particles, except for the standard KCl solution. The equine serum sample was acquired in Sigma-Aldrich (reference H1270–100 ML), while the urine sample was obtained from healthy volunteers (Ethical permit CE062308). The canal water sample was sampled from “El Albujón” (37°42′58″N 0°51′38″W, Region of Murcia, Spain). The standard solution of 0.1 M KCl was purchased in VWR (reference 87898.290). For electrochemical measurements, all the samples were diluted in 10 mM NaCl supporting electrolyte at appropriate ratios to maintain adequate conductivity and to fit within the linear detection range provided by the developed electrochemical protocols. IC was employed as a second analytical technique to analyze K^+^ levels in all the samples. In this case, the serum sample was also deproteinized by mixing four parts of absolute ethanol with one part of serum. This mixture was then centrifuged at 6000 rpm for 10 min in a microcentrifuge model MicroStar 12 provided by VWR.

### Preparation of the K^+^-Selective Electrode

The ITO glass electrode was first cleaned with ethanol by ultrasonication (USC200TH, VWR) and dried with nitrogen flow. A PTFE tape was placed in the ITO surface to delimitate an active circular area with a diameter of 8.00 ± 0.01 mm. An electrical contact was secured using copper tape. Then, a solution comprising 0.1 M of LiClO_4_ and 3-octylthiophene in acetonitrile was prepared for the POT synthesis. This solution was degassed through purging nitrogen for 15 min and a POT film was formed on the ITO surface through electrochemical polymerization conducted via cyclic voltammetry from 0 to 1.5 V at 100 mV/s and 2 scans, ending with a discharge at 0 V for 120 s. A homemade Ag/AgCl wire was used as the reference electrode and platinum rod was employed as counter electrode. Afterward, the synthesized POT film was immersed in ACN for 30 min and then in THF for 10 s to remove any impurity. Finally, the film was carefully dried with nitrogen flow.

Subsequently, the membrane cocktail was deposited on top of the POT film. The corresponding cocktail contained 20 mg of PU, 20 mg of DOS, 0.8 mg of NaTFPB and 2 mg of potassium ionophore I dissolved in 2 mL of THF. A volume of 25 μL of this cocktail was coated (1500 rpm, 120 s) on the ITO-POT electrode using a spin coater model WS-650–23B provided by Laurell Technologies. This procedure allows to obtain a potassium-selective nanomembrane of approximately 230 nm in thickness.^10^ Based on the total amount of cation exchanger (NaTFPB) incorporated, the theoretical ion-exchange capacity of this membrane was estimated to be 21.2 μC. Since the conducting polymer POT is present in excess, its redox switching is stoichiometrically coupled to the incorporation/expulsion of counterions to maintain electroneutrality. Consequently, the maximum charge that can be delivered during electrochemical interrogation is governed by the total amount of TFPB^–^ within the membrane, establishing the upper boundary for ion transfer from the thin-layer sample.

### Description of the Thin-Layer Electrochemical Cell

The electrochemical cell consisted of two PTFE holders designed for electrode placement. The ITO-POT-membrane serving as the working electrode, was placed in the first PTFE holder. The second PTFE holder contained the pseudocounter/reference electrode, which consisted of a homemade Ag/AgCl electrode. The mechanism underlying the analytical concept and the related experimental setup relies on achieving exhaustive ion release/uptake to/from the sample, a process that requires well-defined thin-layer conditions. To this end, the thin-layer compartment for the sample was defined by a rubber spacer (thickness 100 ± 5 μm) placed between the working electrode (ITO-POT-membrane) and the CE-RE and connected in turn with the inlet and outlet of the cell. The entire cell is assembled with screws and nuts and the sample injected with a peristaltic pump. This spacer-based design was intentionally selected for its pragmatic advantages in early stage validation, including low-cost fabrication, reliable sealing, rapid assembly, and straightforward reuse. Although compliant materials may introduce minor variations in the effective cell volume, such variability is minimized by the mechanical constraints of the rigid PTFE housing and a standardized screw-tightening procedure, which together ensure reproducible spacer compression.

The resulting microfluidic configuration offers significant advantages, including sufficient control over the sample volume and the facilitation of rapid and reproducible sample renewal. Furthermore, the confined geometry ensures that the sample volume is sufficiently small toward complete analyte depletion within practical experimental time scales, which is fundamental for a calibration-free quantification. The use of the combined Ag/AgCl counter-reference was also evaluated. Given the 10 mM NaCl background, the Cl^–^ consumption during exhaustive K^+^ uptake (maximum theoretical charge of 21.2 μC determined by the amount of cation exchanger in the membrane) is negligible (<1% in concentration), ensuring a stable reference potential with neglectable influence of Cl^–^ concentration change. Additionally, the common ion effect provided by the chloride background suppresses AgCl solubility to ca. 0.016 μM of Ag^+^, a concentration that does not interfere with the K^+^ signal due to the high selectivity of valinomycin.^21^ A diagram of the microfluidic device containing the electrodes and sample is shown in Figure 1a.

**Figure 1:**
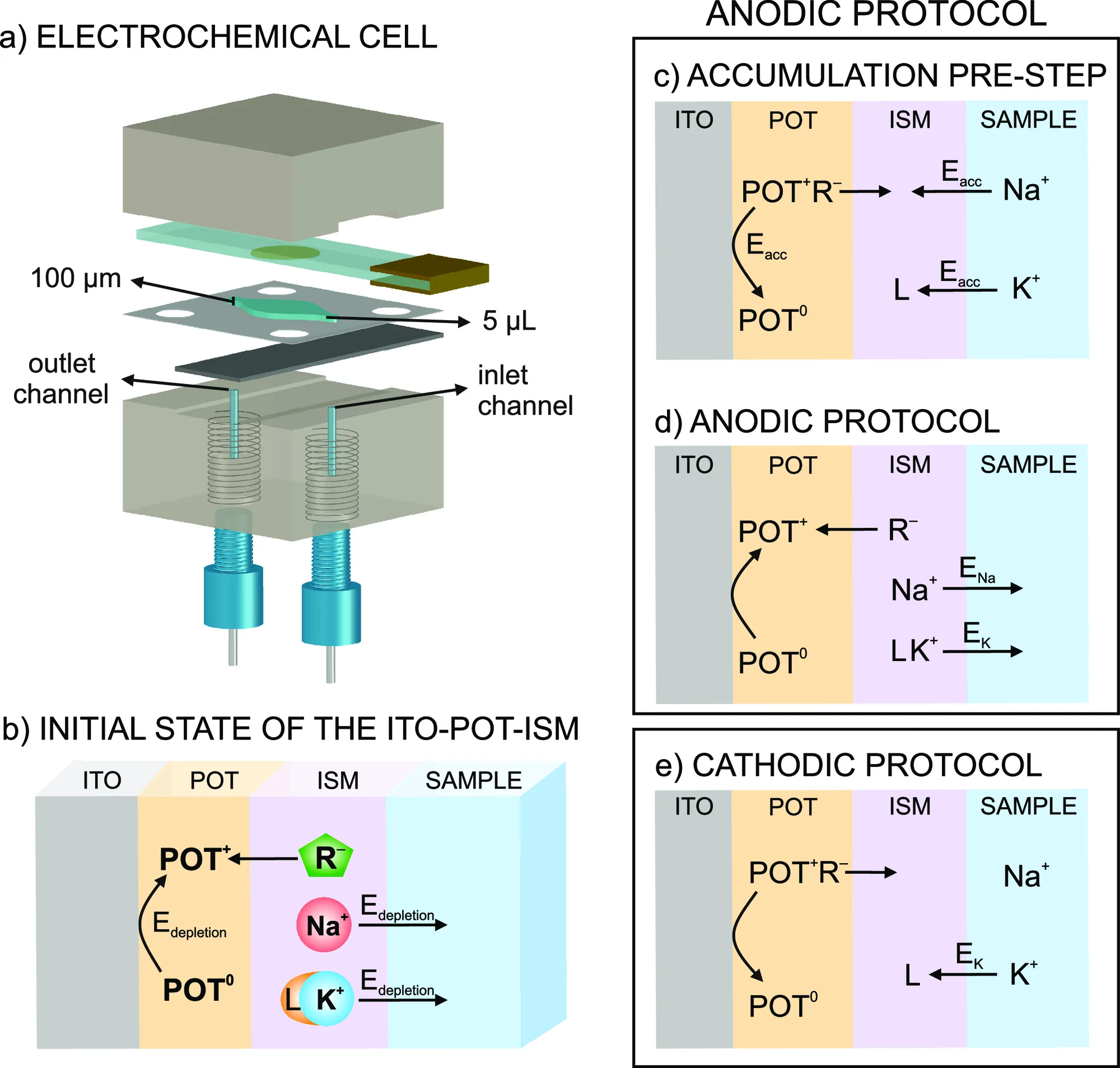
(a) Microfluidic cell designed to confine the sample to a thin-layer domain. (b) Initial state of the ITO-POT-membrane electrode when introducing the sample upon application of 1 V (*E*_depletion_). (c) Accumulation step in the anodic protocol through application of *E*_acc_. (d) Working mechanism of the anodic protocol, applying three consecutive potentials: *E*_baseline_, *E*_Na_ and *E*_K_. (e) Working mechanism of the cathodic protocol. R^–^ = TFPB^–^. L = valinomycin.

## Results and Discussion

### Protocols Defined for the Chrono Electrochemical Measurements

Two different measurement protocols were implemented. They are here termed as anodic- and cathodic-driven ion-transfer (IT) thin-layer coulometry (A-IT-TLC and C-IT-TLC, respectively). Both protocols were initiated with the polarization of the working electrode at 1 V for 150 s, while the sample was introduced into the microfluidic cell using a peristaltic pump at a flow rate of 80 μL/min. The purpose of this step was the cleaning of the membrane from any present cation: at such a potential, POT is completely in its oxidized form doped with the TFPB^–^. Being POT in excess with respect to the cation exchanger NaTFPB, all the TFPB^–^ pairs POT^+^ and Na^+^ is entirely expelled to the sample solution. Figure 1b shows the original state of the ITO-POT-membrane electrode in contact with an aqueous sample considering this first step, with *E*_depletion_ stating for the potential used for the membrane cleaning (1 V).

The A-IT-TLC protocol is summarized in Figure 1c,d. It commences with an accumulation step in which a voltage of −0.2 V is applied for 60 s (*E*_acc_). POT^+^ is reduced to POT^0^ and the dopant anion TFPB^–^ liberates to pair cations entering from the sample, preserving the electroneutrality of the system. Accordingly, all the positive exchangeable positions fixed in the membrane by the TFPB^–^ are occupied by Na^+^ and K^+^, with the ratio between these two cations (and thus the corresponding charge of each one in the membrane) changing with the K^+^ concentration in the sample. Subsequently, three different potentials are sequentially applied for 40 s each: (i) a potential where no ion transfer occurs to use it as the baseline (*E*_baseline_); (ii) a potential to expel the background cation(s) (*E*_Na_, which is Na^+^ in the basic experiments herein performed); and (iii) a potential to selectively transfer K^+^ (*E*_K_). The values of *E*_baseline,_ *E*_Na_ and *E*_K_ are selected based on the peak potentials (*E*_peak_) observed in a previous anodic linear sweep voltammetry (A-LSV, from −0.2 to 1.2 V at a scan rate of 50 mV s^–1^) experiment (see Figure S1 in the Supporting Information).

In this kind of system where electron transfer is coupled with ion transport and structural rearrangements of the redox-active element, *E*_peak_ does not represent a purely kinetic parameter. Instead, it reflects a convolution of charge-transfer kinetics, ohmic drop, and mass-transport contributions.^22^ Furthermore, while efficient coulometry for simple dissolved redox species often requires stepping well into the mass-transport-limited plateau, this principle is not often applicable to systems involving ion insertion into condensed phases. In such architectures, driving the potential significantly beyond *E*_peak_ can induce excessive overpotentials, parasitic reactions, and nonfaradaic contributions, ultimately compromising Coulombic efficiency and material integrity.^23^ Accordingly, the operating potentials used in this work were deliberately selected to achieve exhaustive charge recovery while avoiding these nonideal effects and maintaining a balance between reaction rate and system stability. Notably, the associated voltammogram must show two peaks, with the first one corresponding to Na^+^ (or any other cation different from K^+^) expelling from the membrane, and the second one to the targeted K^+^ release.

Upon progression of the applied potential in the anodic direction, the neutral POT^0^ is oxidized to POT^+^ causing the counterion TFPB^–^ to migrate toward the POT film for doping, and consequently, the cations present in the membrane are expelled to the sample solution. Initially, the potential is insufficient to drive K^+^ transfer, and only the background cation (Na^+^) is released, producing the first peak. Since K^+^ is strongly retained in the membrane due to the ionophore, its transfer occurs at a higher potential. These two transfers define two potential regions associated with *E*_Na_ and *E*_K_, with *E*_baseline_ being selected in a zone where no faradaic process is detected. The current dynamic profiles obtained upon successive application of *E*_baselineL,_ *E*_Na_ and *E*_K_ are integrated to determine the charge associated with each event.

Notably, prior to charge calculation, several signal corrections were applied to isolate the faradaic charge. In chronoamperometry, an offset correction was first applied to align the signal to zero at the end of the pulse. Then, the contribution of nonfaradaic processes was accounted for by subtracting the response obtained at *E*_baseline_, where no IT occurs. Finally, a blank subtraction was performed to remove any residual contribution arising from trace levels of K^+^ in the electrochemical cell. Figure S2 in the Supporting Information depicts an example of this data treatment procedure. In LSV, a baseline correction was used to compensate for capacitive contributions by defining a linear baseline from the voltammetric response. When the voltammogram displayed partially overlapping peaks, Gaussian fitting was employed for peak deconvolution. The charge associated with each peak was calculated by integrating the fitted curves and dividing by the scan rate (see Figure S3 for an example). As in chronoamperometry, a final blank subtraction was applied to eliminate residual contributions from K^+^ present in the background solution or in the electrochemical cell.

The C-IT-TLC protocol is illustrated in Figure 1e. The procedure starts with the POT in its oxidized form POT^+^ doped with the TFPB^–^. Then, two potentials are sequentially applied for 40 s each: (i) a potential at which no ion transfer occurs to use it as baseline (*E*_baseline_), and (ii) a potential that promotes direct uptake of K^+^ from the aqueous phase into the membrane (*E*_K_). These potentials are identified from a previous cathodic linear sweep voltammetry (C-LSV) that is represented in Figure S4. The cathodic voltammogram is recorded from 1.1 to −0.1 V at a scan rate of 5 mV/s. The slow scan rate is necessary to allow sufficient time for cation accumulation within the membrane, yielding two well-defined peaks. Essentially, as the potential shifts in the cathodic direction, the oxidized POT^+^ species are progressively reduced to POT^0^, prompting the expulsion of TFPB^–^ anions from the polymer film to the membrane. This process generates a charge imbalance in the membrane that is compensated by the incorporation of cations from the solution.

Under the selective conditions provided by the ionophore, K^+^ is preferentially accumulated over background cations (Na^+^), enabling its transfer into the membrane without the prior accumulation of any background cation, in contrast to the A-IT-TLC case. Such mechanistic asymmetry justifies the use of different scan rates for cathodic (5 mV/s) and anodic (50 mV/s) protocols, which is a deliberate and standard practice when ion insertion and deinsertion processes exhibit distinct dynamics. In these architectures, voltammetric responses reflect coupled kinetics and transport phenomena rather than a simple transition to mass-transport control.^24,25^ Consequently, the scan rate was adjusted to ensure that each process is characterized under its optimal regime, accounting for the inherent mechanistic differences between the two operating modes.^26^ The current–time curves obtained upon application of *E*_K_ are integrated after baseline correction to calculate the charge associated with each process. Nonfaradaic contribution was corrected by subtracting the current profile recorded at *E*_baseline_ from that obtained at *E*_K_, yielding the net charge associated with the K^+^ transfer into the membrane. Similar as in the A-IT-TLC mode, if there is any signal associated with residual K^+^, it is removed from all curves to ensure that each measurement begins from a clean baseline.

### Investigation of Anodic Protocols with the ITO-POT-Membrane Electrode in the Thin-Layer Cell

The A-IT-TLC protocol was evaluated by progressively increasing the K^+^ concentration in the thin-layer sample (volume of 5.0 ± 0.3 μL) in the range of 1–50 μM in 10 mM NaCl background electrolyte. During the anodic sweep potential, the oxidation of the conducting polymer (POT^0^ → POT^+^) triggers the expulsion of cations from the membrane into the solution. This process is reflected in the experimental voltammogram with two distinct peaks revealing the sequential transfer of Na^+^ and K^+^, as observed in Figure 2a. Initially, in absence of K^+^ in the solution, the membrane only contains Na^+^ and a single peak was observed at ca. 516 mV, corresponding to Na^+^ transfer from the membrane to the solution. As K^+^ is introduced into the thin-layer sample, this Na^+^ peak progressively decreased while a second peak emerged at 762 mV associated with K^+^ release from the membrane to the solution. As expected, the K^+^ peak appeared at a higher potential than the Na^+^ peak, reflecting the greater energy required to release K^+^ from the membrane due to the formation of a stable complex with the selective ionophore.^15^ When a sufficiently high K^+^ concentration (e.g., 50 μM) is present in the sample, the Na^+^ signal disappears entirely, indicating that the membrane is saturated with K^+^. This behavior implies that all available positive vacancies in the membrane, which were initially occupied by Na^+^ (charge of 20.5 μC) were completely replaced by K^+^ (charge of 20.6 μC). These maximum charges are in good agreement with the theoretical exchange capacity of the membrane (ca. 21.2 μC), as estimated from the total amount of cation exchanger present in it (NaTFPB). Importantly, the ionic conductivity of the membrane is preserved throughout this ion-exchange process due to the constant concentration of fixed ionic sites, which ensures electroneutrality and charge transport even under conditions of exhaustive analyte depletion in the thin-layer sample.

**Figure 2:**
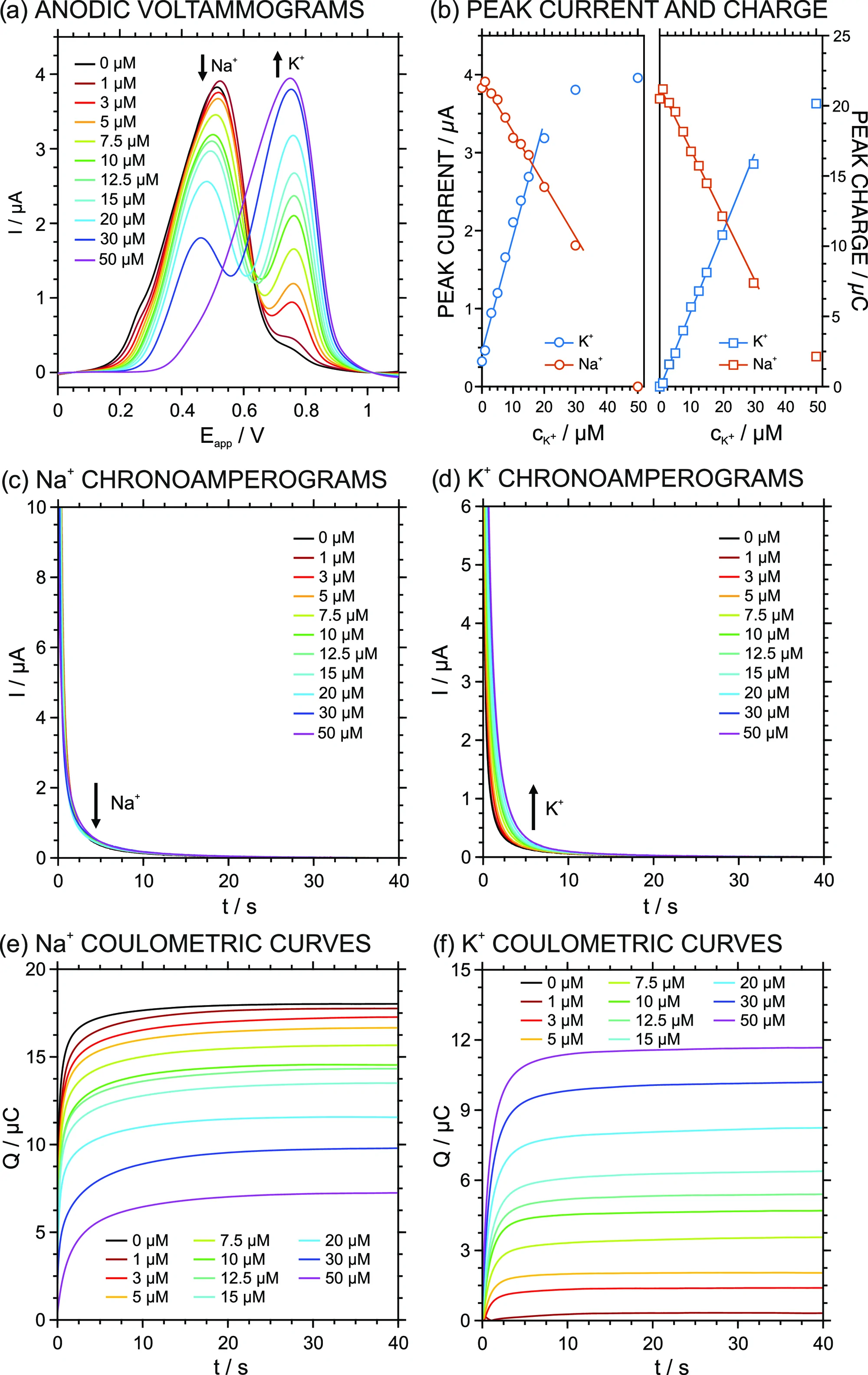
(a) Anodic LSVs at increasing KCl concentrations in 10 mM NaCl, scan rate = 50 mV s^–1^. (b) Plot of the peak currents and charges for Na^+^ and K^+^ transfers versus the K^+^ concentration in the sample solution, obtained from the LSV data. (c) Chronoamperograms for Na^+^ at increasing KCl concentrations in 10 mM NaCl applying *E*_Na_ = 500 mV. (d) Chronoamperograms for K^+^ at increasing KCl concentrations in 10 mM NaCl applying *E*_K_ = 750 mV. (e) Corrected chronocoulometric curves for Na^+^ at increasing KCl concentrations in 10 mM NaCl. (f) Corrected chronocoulometric curves for K^+^ at increasing KCl concentrations in 10 mM NaCl.

Peak current and charge trends with the K^+^ concentration in the sample solution are presented in Figure 2b. Notably, a minor peak at 765 mV was observed in the background voltammogram, suggesting some K^+^ contamination in the sample solution and/or the electrochemical cell. This residual signal was removed in the final signal treatment as described in the Experimental Section. Also, the Na^+^ peak displayed a shoulder around 264 mV, which could be attributed to electrochemical and structural heterogeneity within the POT film. This behavior, commonly observed in polythiophene derivatives, reflects multiple redox transitions associated with different states of the polymer backbone, leading to a distribution of oxidation potentials rather than a single well-defined peak.^27,28^ A linear correlation was observed for the peak current and charge with K^+^ concentration, with values extracted from baseline-corrected voltammograms: *I*_K_^+^ (μA) = 0.1475 *c*_K_^+^ (μM) + 0.4493; *R* ^2^ = 0.9833 (linear range of response, LRR: 1–20 μM) and *Q*_K_^+^ (μC) = 0.5379 *c*_K_^+^ (μM) – 0.0491; *R* ^2^ = 0.9985 (LRR: 1–30 μM). The charge response exhibited a wider linear range and stronger correlation with K^+^ concentration than that observed for the peak current. Notably, peak current reflects instantaneous signal values and is more susceptible to distortions from double-layer charging or uncompensated resistance, whereas charge integration captures the total faradaic response over time.

The presence of well-resolved peaks in the A-LSV experiment allows for the selection of appropriate potentials for the chronoamperometric sequence: *E*_baseline_ = 100 mV, *E*_Na_ = 500 mV and *E*_K_ = 750 mV. Thresholds of 10% for *E*_Na_ and <1% for *E*_K_ were considered negligible to have a significant impact on the chronoamperometric response. Sequential application of these steps yielded the current–time curves shown in Figures S5, and 2c,d for *E*_baseline_, *E*_Na_ and *E*_K_, respectively. The dynamic (accumulative) integration of the signals related to *E*_Na_ and *E*_K_ results in the charge profiles corresponding to Na^+^ and K^+^ transfers (Figure 2e,2f). Notably, the charge associated with nonfaradaic events was calculated from the integration of the *E*_baseline_ pulse to be within the range of 3.5–4.0 μC. An increment in the charge for K^+^ while Na^+^ charge decreases was obtained at increasing K^+^ concentration in the sample solution. As shown in Figure 2d, the current effectively decayed to the baseline, while the integrated charge (Figure 2f) reached a stable plateau before the end of the pulse for all tested concentrations. A quantitative analysis of the current stability during the last 10 s (from 30 to 40 s) revealed slopes ranging from 0.57 to 1.12 nA s^–1^. Such minimal variation corresponds to an estimated error of 0.03–1.40% in the concentration calculation, indicating that exhaustive analyte depletion is achieved within the experimental time frame.

Figure 3 shows the evolution of the total K^+^ charge obtained in the chronoamperometric experiment at 40 s with its concentration in the sample solution. In the plot, three equally prepared electrodes are considered (the corresponding chronocoulograms are presented in Figure S6). A good linearity from 1 to 30 μM was revealed, following the equation: *Q*_K_^+^ (μC) = 0.3403 *c*_K_^+^ (μM) + 0.6016; *R* ^2^ = 0.9758. Moreover, the between-electrode reproducibility was quantified by the standard error of the slopes (0.02 μC μM^–1^), corresponding to a relative standard deviation (RSD) of 5.9%, which is noteworthily good given the manual steps involved in their preparation. Interestingly, the upper limit of this linear range approaches the maximum concentration that the membrane can accommodate considering total depletion of K^+^, i.e., its total exchange capacity imposed by the amount of the cation exchanger in the membrane (21.2 μC in terms of charge, 44 μM in terms of concentration). Consequently, at higher concentrations, the system reaches a plateau because the available ion-exchange sites are fully occupied. Notably, the linear range was wider (up to 30 μM) than that obtained from the peak current in LSV (up to 20 μM).

**Figure 3:**
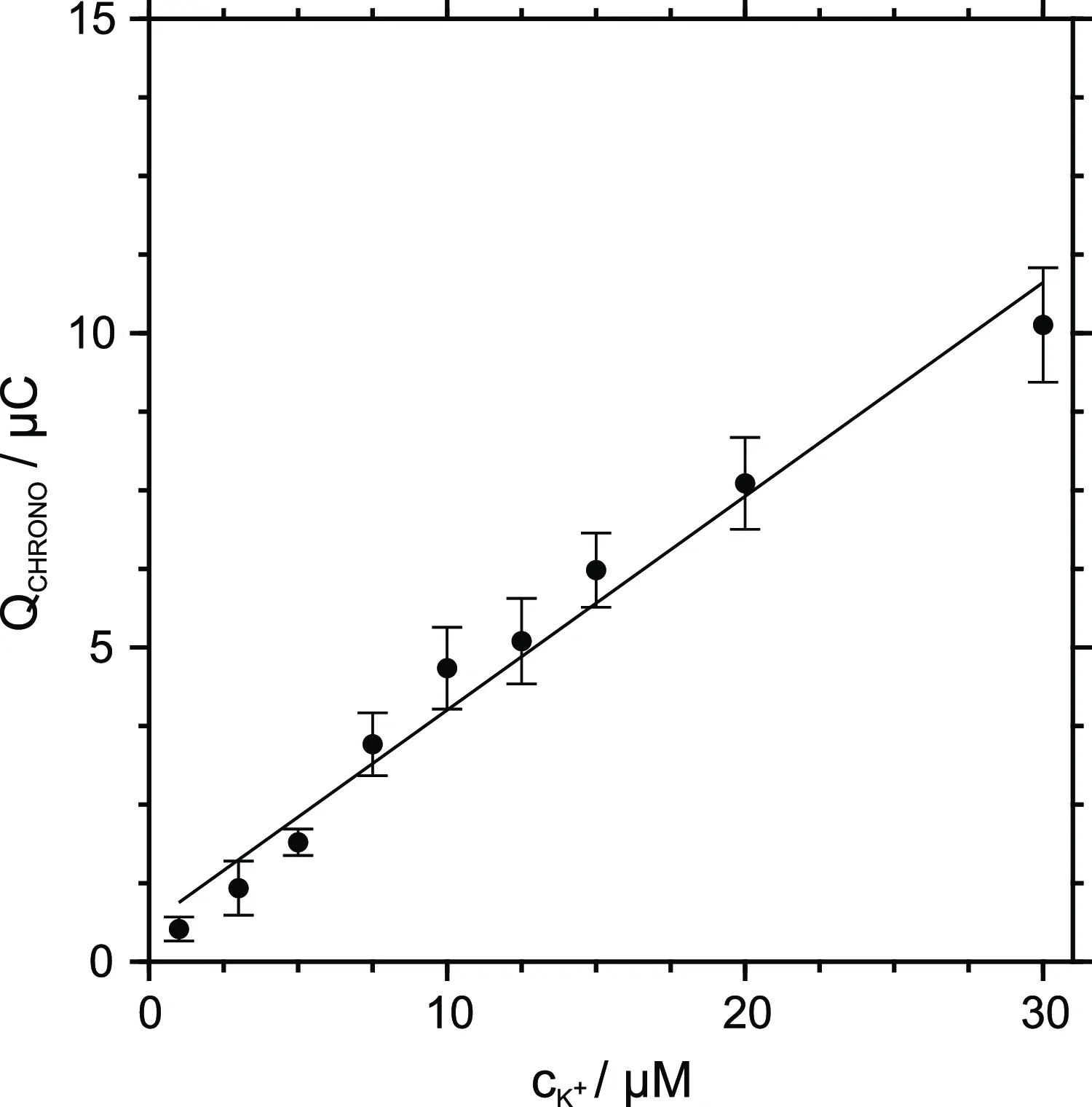
Plot of the averaged K^+^ charge obtained from A-IT-TLC experiments versus the K^+^ concentration in the sample solution (*n* = 3 electrodes).

In comparison with the peak charge calculated from the LSV, chronoamperometry displayed lower sensitivity and slightly reduced correlation coefficient, while still maintaining a wider linear range. The application of a constant potential step in chronoamperometry allows for a sufficiently long time frame (40 s) to ensure that the analyte is completely depleted. This is evidenced by the current decay reaching a stable background. In contrast, the dynamic nature of LSV may lead to incomplete depletion if the scan rate is not sufficiently slow to allow for the exhaustive transfer of the analyte. Also, chronoamperometry offers simpler signal treatment and greater operational stability, making it a valuable complementary technique to LSV. Nevertheless, in terms of calibration-free possibilities via faradaic quantification, a deep analysis will be provided below considering a systematic comparison of all the protocols herein established and tested.

### Investigation of Cathodic Protocols with the ITO-POT-Membrane Electrode in the Thin-Layer Cell

The C-IT-TLC protocol was studied by increasing the K^+^ concentration in the range of 1–50 μM in 10 mM NaCl background solution. The working electrode was first interrogated under LSV in the potential window from 1.1 to −0.1 V (Figure 4a). When the potential is swept in the cathodic direction, the POT^+^ is reduced to POT^0^ and the cations are driven to enter the membrane translating into two distinct peaks: one for K^+^ and the other for the rest of cations in the sample solution (Na^+^ in this experiment). The evolution of peak current and charge with the K^+^ concentration in the sample solution are presented in Figure 4b. For the background solution only one peak at ca. 450 mV was observed, associated with the Na^+^ uptake into the membrane. Once K^+^ was introduced in the thin-layer sample, the Na^+^ peak gradually decreased while a second peak appeared and increased at 0.65 V. This relates to the K^+^ entry into the membrane. While the K^+^ peak was not well-defined at the lowest concentration (1 μM), well-resolved peaks for both Na^+^ and K^+^ were observed at higher concentrations. Then, at the highest concentration tested (50 μM), only one peak was found due to the membrane saturation with K^+^. In essence, the positive sites in the membrane, which were first occupied by Na^+^ (charge of 15.4 μC) are totally replaced by K^+^ (charge of 15.8 μC) from that concentration.

**Figure 4:**
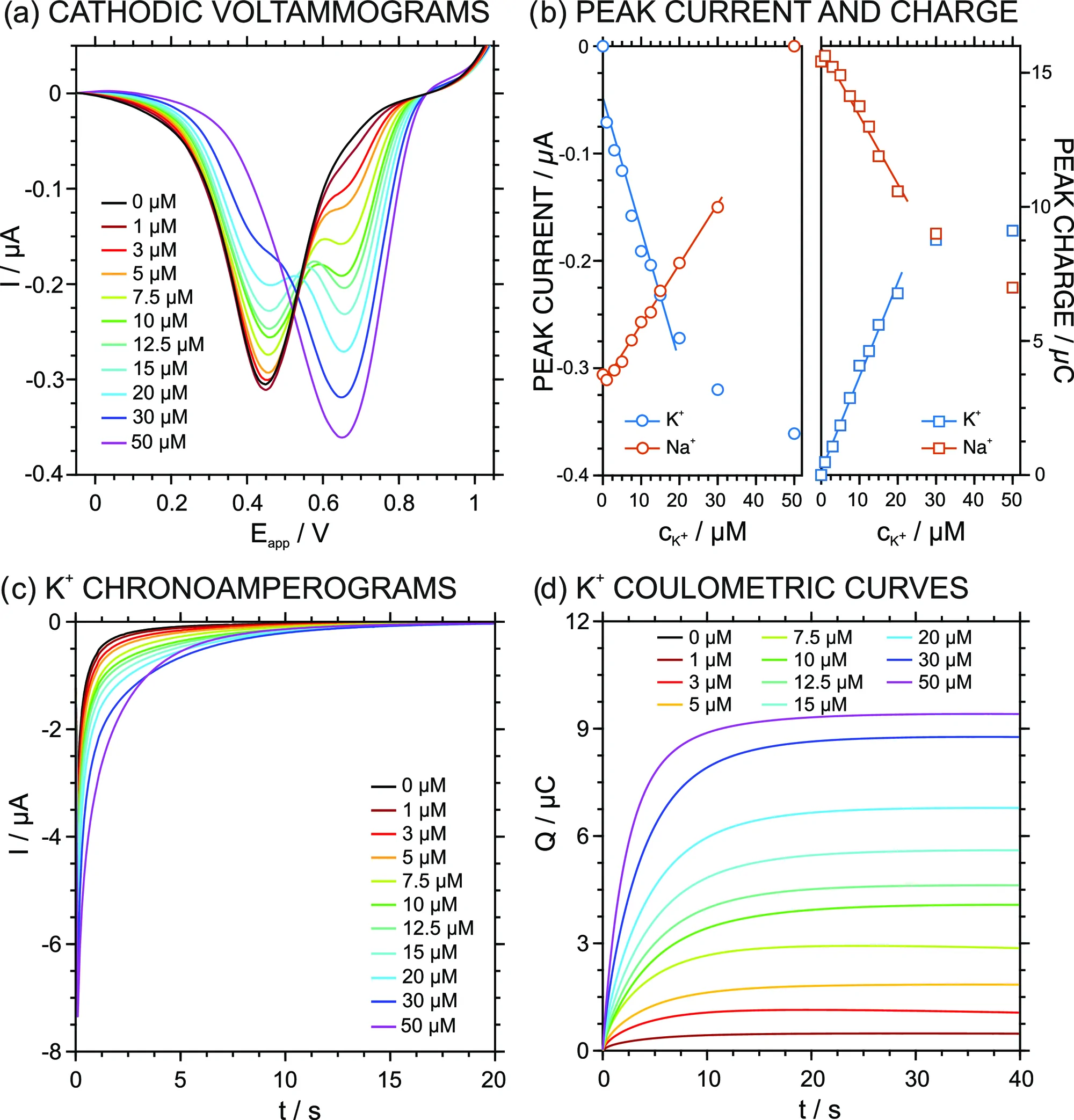
(a) Cathodic LSVs at increasing KCl concentrations in 10 mM NaCl, scan rate = 5 mV s^–1^. (b) Plot of the peak currents and peak charges for Na^+^ and K^+^ versus the K^+^ concentration. (c) Chronoamperograms for K^+^ at increasing KCl concentrations in 10 mM NaCl. (d) Corrected chronocoulometric curves for K^+^ at increasing KCl concentrations in 10 mM NaCl.

A certain linear correlation was observed for both the peak current and charge with the K^+^ concentration following the equations: *I*_K_^+^ (μA) = −0.0108 *c*_K_^+^ (μM) – 0.068, *R* ^2^ = 0.9834 (LRR: 1–20 μM) and *Q*_K_^+^ (μC) = 0.3903 *c*_K_^+^ (μM) – 0.0057, *R* ^2^ = 0.9977 (LRR: 1–30 μM). The peak charge response demonstrated a slightly broader linear range (up to 30 μM) and a stronger correlation with K^+^ concentration (*R* ^2^ = 0.9977) than the peak current (up to 20 μM, *R* ^2^ = 0.9834). This better performance can be ascribed to the integrative nature of charge measurements, which enhances the signal-to-noise ratio. Conversely, peak current represents instantaneous signal values, making it more vulnerable to distortions from uncompensated resistance. Moreover, while capacitive current is indeed integrated into the total charge, this contribution is systematically accounted for and removed through the background subtraction of the 10 mM NaCl blank, ensuring that the final analytical signal corresponds exclusively to the faradaic process.

The voltammetric results served to select *E*_baseline_ and *E*_K_ to be applied in the chronoamperometry measurements: 950 and 650 mV, respectively. A potential fluctuation of less than 1% was considered negligible to have a significant impact on the chronoamperometric response. The potentials *E*_baseline_ and *E*_K_ were sequentially applied, displaying the curves in Figures S7 and 4c, respectively, for increasing K^+^ concentrations in the sample. The integration of the current signal from the chronoamperograms yielded the corresponding charge–time presented in Figure 4d, which revealed increasing charges as K^+^ transfer increases with the concentration in the sample. In this sense, Figure 5 depicts the charge related to the K^+^ transfer with its concentration obtained from the experiment of Figure 4c sampled at 40 s. This plot represents the average of three identically prepared electrodes, with their corresponding coulometric curves provided in Figure S8. The system exhibited excellent linearity from 1 to 20 μM following the equation: *Q*_K_^+^ (μC) = 0.3307 *c*_K_^+^ (μM) + 0.1966, *R* ^2^ = 0.9899. Furthermore, adequate between-electrode reproducibility was confirmed by the standard error of the slope (0.01 μC μM^–1^), which corresponds to an RSD of 3.0%.

**Figure 5:**
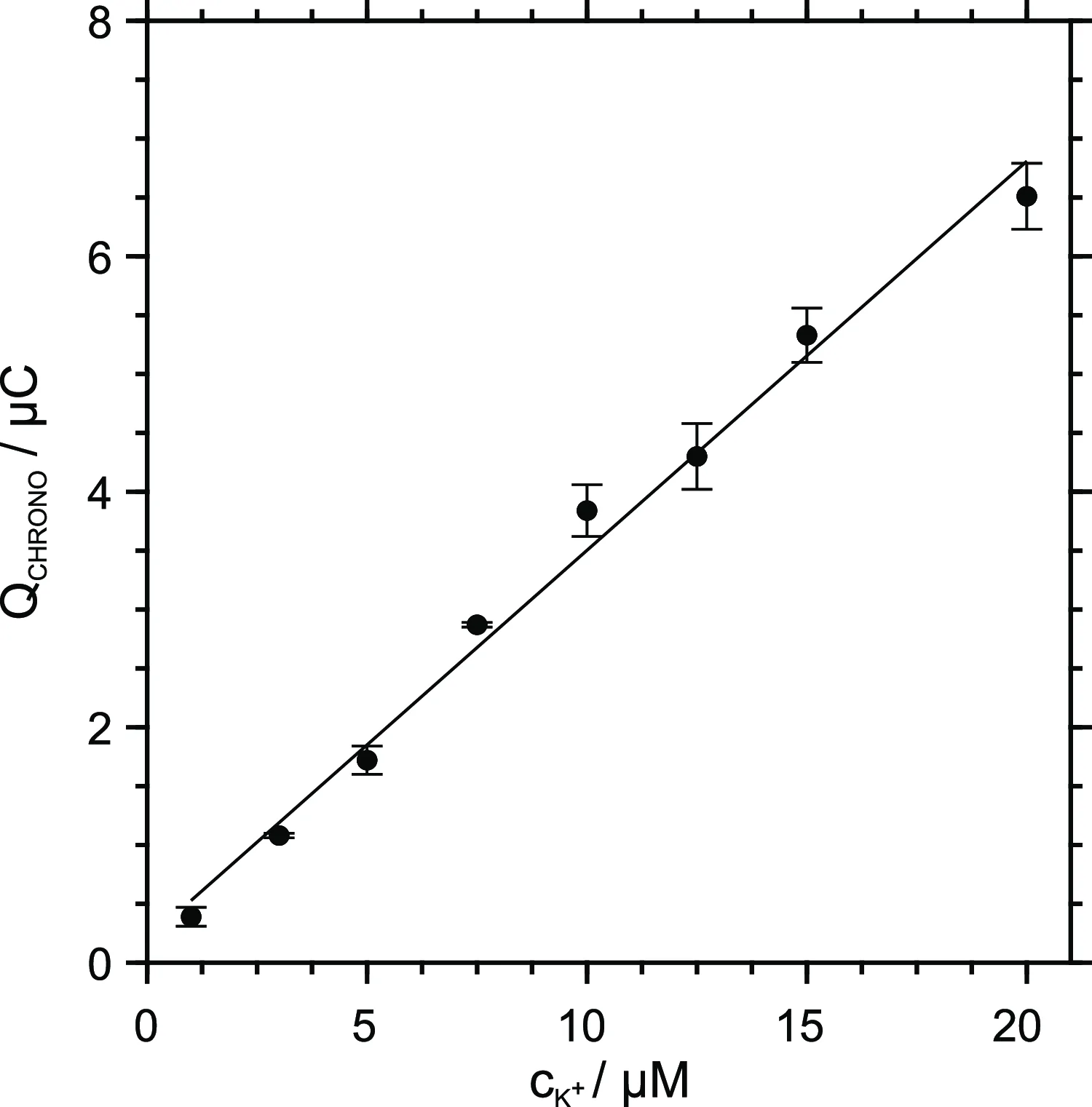
Plot of the averaged K^+^ charge obtained from C-IT-TLC experiments versus the K^+^ concentration in the sample solution (*n* = 3 electrodes).

Compared to LSV data, C-IT-TLC provided comparable sensitivity of 0.3307 μC μM^–1^ (vs 0.3903 μC μM^–1^ for LSV), but a slightly narrower linear range extending up to 20 μM instead of the 30 μM observed in LSV, while still maintaining high linearity (*R* ^2^ = 0.9899). LSV measurements require significantly longer analysis because of the slow scan rate required to properly visualize the voltammetric peaks (5 mV s^–1^). For this reason, combined with the simpler data treatment, chronoamperometry stands up as the preferred method. When compared to the A-IT-TLC protocol, the C-IT-TLC approach exhibits a sharper linear range (1–20 μM vs 1–30 μM). Indeed, each protocol may reveal a distinct linear range of response because of the different ion-transfer events they involve. While A-IT-TLC relies on ion accumulation in the membrane followed by expulsion to the sample solution, C-IT-TLC is governed by direct ion uptake from the sample to the membrane. The latter offers shorter measurement times, whereas the anodic mode requires the prior expulsion of background cations before analyte transfer can occur. In any case, the close agreement in performance supports the reliability of the coulometric readout and highlights the complementary nature of both approaches for monitoring ion-transfer dynamics within the membrane.

### Investigation of the Analytical Performance of the Anodic and Cathodic Protocols

Since this is the first time that pulsed, electrochemical techniques are applied to ISEs based on nanometer-sized membranes connected to thin-layer samples, the repeatability of the A-IT-TLC and C-IT-TLC protocols was evaluated: triplicate experiments were conducted using the same electrode but performing each of the protocols. Two K^+^ concentrations within the respective LRR were selected (1 and 10 μM KCl), focusing the discussion on the K^+^ response (i.e., the one that will be exploited for analytical purposes). As shown in Figure S9 (Supporting Information), the coulometric and amperometric responses for both concentrations demonstrated a strong overlap across replicates. For the C-IT-TLC protocol, the RSDs were 1.8% and 1.3% for 1 and 10 μM, respectively, while the A-IT-TLC protocol showed RSDs of 5.2% and 0.8%, respectively. Although the anodic method displayed slightly higher variability at the lower concentration, the standard deviations related to measured charges remained low (<0.2 μC) across all conditions, confirming the good repeatability of both methods (see Table S1 in the Supporting Information).

The reversibility was also investigated, interrogating the ITO-POT-membrane electrode with successive cycles: increasing and decreasing the concentration, following the sequence of 1, 10, 1, 10, 1, and 10 μM KCl. As observed in Figure S10 (Supporting Information), the C-IT-TLC protocol demonstrated excellent reversibility with standard deviations <0.06 μC and corresponding RSD values <2% (see Table S2 in the Supporting Information). In contrast, the A-IT-TLC protocol, as in the case of repeatability, exhibited higher variability. This was particularly relevant at the lower concentration (1 μM KCl), with an RSD of 8.7%. This worsening in reversibility can be attributed to the increased complexity of the protocol, involving both an accumulation step and the previous Na^+^ expelling pulse, which may introduce intrinsic experimental variations affecting the reversibility of the protocol, especially when detecting small K^+^ concentrations.

Tables 1 and 2 show the parameters associated with the analytical performance of the anodic and cathodic protocols from a coulometric perspective, including the added and calculated average concentrations determined via the Faraday Law, measured average charges, coulometric efficiencies (defined as the ratio between the experimentally measured charge and the theoretical charge expected from Faraday’s law, considering the added concentration and the sample volume) and the relative errors regarding the added concentrations. Extended raw data obtained from three identical ITO-POT-ISM electrodes using the A-IT-TLC and C-IT-TLC protocols are provided in the Supporting Information (Tables S3 and S4, respectively).

**Table 1:** Coulometric K^+^ Detection Using the A-IT-TLC Protocol

*c*_K_^added^ (μM)	*Q*_K_^chrono^ (μC)^a^	*c*_K_^chrono^ (μM)^a^	efficiency (%)^b^	relative error (%)^c^	*Q*_K_^LSV^ (μC)^a^	*c*_K_^LSV^ (μM)^a^	efficiency (%)^b^	relative error (%)^c^
1.0	0.52 ± 0.19	1.07 ± 0.39	107.4	7.4	0.27 ± 0.04	0.58 ± 0.11	57.6	42.4
3.0	1.17 ± 0.43	2.42 ± 0.89	80.5	19.5	1.38 ± 0.20	2.84 ± 0.41	94.8	5.2
5.0	1.90 ± 0.20	3.92 ± 0.42	78.5	21.5	2.31 ± 0.15	4.77 ± 0.31	95.5	4.5
7.5	3.46 ± 0.50	7.15 ± 1.02	95.3	4.7	3.97 ± 0.04	8.20 ± 0.09	109.3	9.3
10.0	4.67 ± 0.65	9.64 ± 1.35	96.4	3.6	5.62 ± 0.17	11.61 ± 0.35	116.1	16.1
12.5	5.10 ± 0.68	10.53 ± 1.40	84.2	15.8	6.80 ± 0.02	14.04 ± 0.04	112.3	12.3
15.0	6.23 ± 0.59	12.86 ± 1.21	85.7	14.3	8.12 ± 0.02	16.77 ± 0.05	111.8	11.8
20.0	7.61 ± 0.73	15.72 ± 1.51	78.6	21.4	10.63 ± 0.22	21.94 ± 0.45	109.7	9.7
30.0	10.13 ± 0.91	20.92 ± 1.88	69.7	30.3	15.27 ± 0.58	31.53 ± 1.20	105.1	5.1
50.0	11.26 ± 1.01	23.24 ± 2.08	46.5	53.5	21.60 ± 1.62	44.59 ± 3.35	89.2	10.8

a The K^+^ concentrations were calculated from the chronoamperogram charge through Faraday Law. Average ± SD (*n* = 3 electrodes).

b The coulometric efficiency was estimated from the known K^+^ concentration added to the sample.

c The relative error in the calculation of the K^+^ concentration was calculated considering that the methodology should present 100% coulometric efficiency.

**Table 2:** Coulometric K^+^ Detection Using the C-IT-TLC Protocol

*c*_K_^added^ (μM)	*Q*_K_^chrono^ (μC)^a^	*c*_K_^chrono^ (μM)^a^	efficiency (%)^b^	relative error (%)^c^	*Q*_K_^LSV^ (μC)^a^	*c*_K_^LSV^ (μM)^a^	efficiency (%)^b^	relative error (%)^c^
1.0	0.39 ± 0.08	0.80 ± 0.17	80.0	20.0	0.32 ± 0.15	0.63 ± 0.42	63.0	37.0
3.0	1.08 ± 0.02	2.22 ± 0.04	74.4	25.6	0.95 ± 0.21	1.96 ± 0.43	65.4	34.6
5.0	1.73 ± 0.12	3.56 ± 0.25	71.6	28.4	1.59 ± 0.14	3.29 ± 0.29	65.8	34.2
7.5	2.87 ± 0.03	5.93 ± 0.05	79.4	20.6	2.71 ± 0.40	5.59 ± 0.82	74.5	25.5
10.0	3.84 ± 0.22	7.93 ± 0.46	79.7	20.3	3.36 ± 0.48	6.94 ± 0.99	69.4	30.6
12.5	4.30 ± 0.28	8.88 ± 0.57	71.3	28.7	4.46 ± 0.21	9.21 ± 0.43	73.7	26.3
15.0	5.33 ± 0.24	11.00 ± 0.49	73.7	26.3	6.02 ± 0.56	12.44 ± 1.16	82.9	17.1
20.0	6.51 ± 0.28	13.44 ± 0.57	67.5	32.5	8.21 ± 0.22	16.95 ± 0.45	84.8	15.2
30.0	8.26 ± 0.50	17.05 ± 1.02	57.1	42.9	10.90 ± 0.61	22.51 ± 1.26	75.0	25.0
50.0	9.68 ± 0.58	19.99 ± 1.20	40.1	59.9	13.84 ± 0.74	28.58 ± 1.53	57.2	42.8

a The K^+^ concentrations were calculated from the chronoamperogram charge through Faraday Law. Average ± SD (*n* = 3 electrodes).

b The coulometric efficiency was estimated from the known K^+^ concentration added to the sample.

c The relative error in the calculation of the K^+^ concentration was calculated considering that the methodology should present 100% coulometric efficiency.

Among the four tested techniques, the A-IT-TLC LSV approach showed the lowest relative errors, generally below 20%, except for the lowest concentration evaluated. This discrepancy could be attributed to residual K^+^ present in the microfluidic device, that may influence the accuracy of the measurement in this regime. Importantly, this method yielded an average coulometry-efficiency of 101 ± 18% across the range 1–30 μM. In contrast, chronoamperometry exhibited a slightly lower efficiency of 86 ± 11%. For the C-IT-TLC protocol, both LSV and chronoamperometry resulted in notably lower errors but also lower efficiencies, with average values around 73 ± 7%. This confirms certain discrepancies between anodic and cathodic methods that are rooted in the contrasting perspectives on K^+^ transport at the membrane–sample interface, viewed either as a release from or an uptake into the membrane, respectively.

The reported average relative errors are influenced by the consideration of the entire concentration range (1–50 μM), where both the lowest and highest concentration levels are more susceptible to experimental variability, or a narrower one. When evaluated over the full range, the average relative errors are 19.2 ± 14.7% (A-Chrono), 12.7 ± 11.1% (A-LSV), 30.5 ± 12.4% (C-Chrono), and 28.8 ± 8.7% (C-LSV). However, these values are strongly affected by the extremes of the concentration range. At low concentrations, small experimental inaccuracies during solution preparation or residual background contributions have a larger impact, whereas at higher concentrations, the finite ion-exchange capacity of the membrane may limit full charge recovery. When the optimal working ranges of each protocol are considered, the relative errors are significantly reduced to 14.4 ± 7.5% (3–20 μM, A-Chrono), 9.4 ± 3.9% (3–50 μM, A-LSV), 25.3 ± 4.6% (1–20 μM, C-Chrono) and 26.1 ± 7.2% (3–30 μM, C-LSV). These results, which are consistent with a volumetric uncertainty of approximately 10%, highlight that the observed discrepancies are primarily governed by practical experimental constraints rather than intrinsic limitations of the coulometric principle.

To evaluate the suitability of the protocols for calibration-free quantification of K^+^, the theoretical charge (*Q*_theo_) was correlated with the experimentally calculated average charge (*Q*_calc_) obtained from three independent electrodes (*n* = 3). Figure 6 presents all possible correlations considering the four approaches from which coulometric data can be obtained: (a) A-IT-TLC chronoamperometry, (b) C-IT-TLC chronoamperometry, (c) A-IT-TLC LSV, and (d) C-IT-TLC LSV. The error bars in each plot represent the standard deviations between replicates, providing an indication of the reproducibility and accuracy of each method. The blue dashed lines represent the ideal slope of unity (*Q*_calc_ = *Q*_theo_), framed by a blue shaded region indicating a ± 10% error band. This band is intended to reflect the cumulative uncertainty inherent to the system. Specifically, a 5% tolerance in the spacer thickness translates into an estimated 5–6% variability in the effective volume, while an additional 3% arises from spacer deformation during assembly (as specified by manufacturer). Other factors include 2–3% associated with lateral diffusion and mass transport limitations,^29^ and 1–2% stemming from variability in experimental handling and cell assembly (Type B contributions to a nonideal behavior).^30,31^ Altogether, these factors result in an overall uncertainty in the range of 11–14%. Therefore, the ±10% band is adopted as a realistic and conservative benchmark for assessing the agreement between experimental and theoretical charge values. Furthermore, the linear fit of the experimental data (solid black line) is presented with its own 10% variation (gray shaded area).

**Figure 6:**
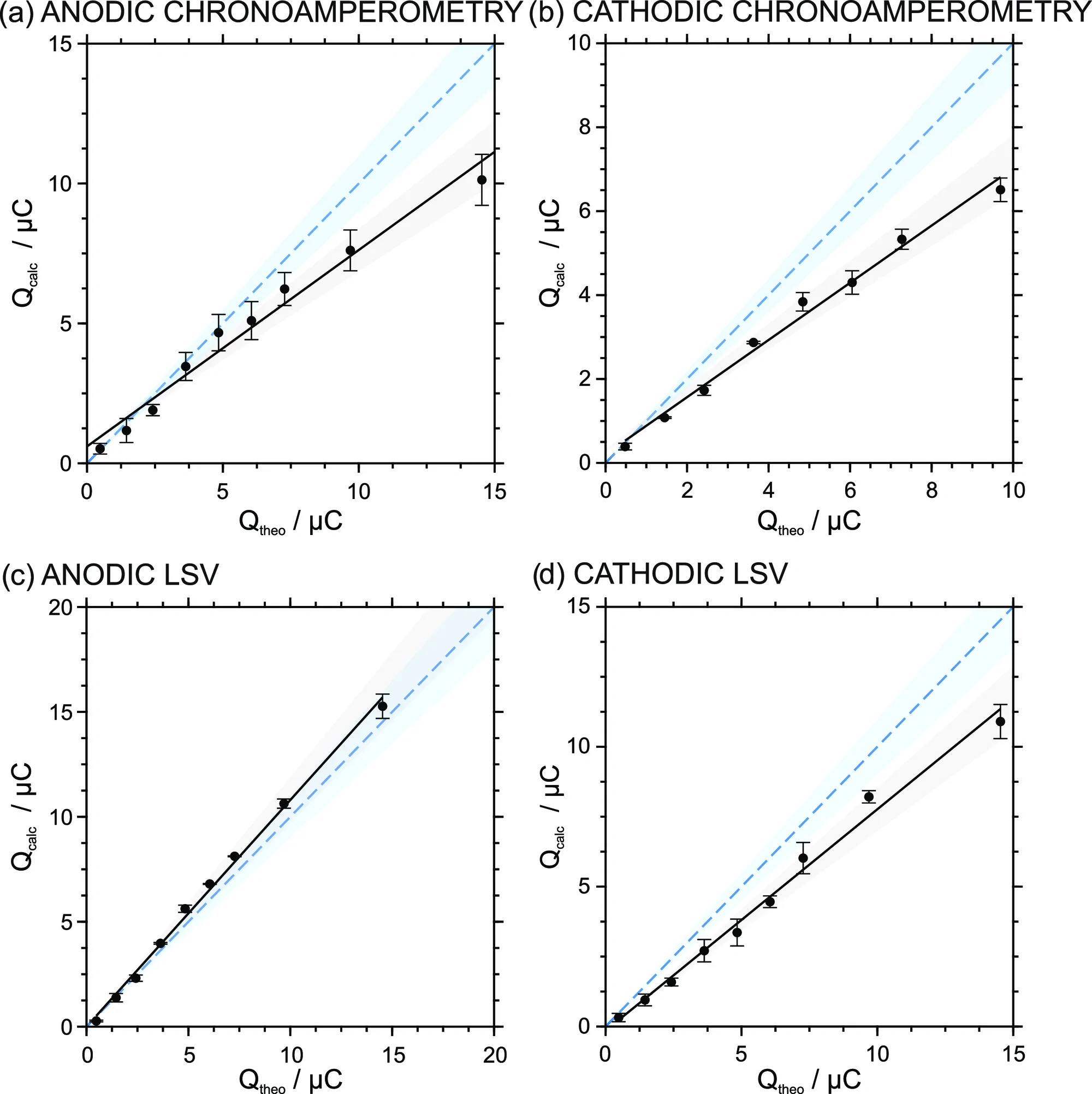
Correlation plots between the theoretical charges (*Q*_theo_) and the average calculated charges (*Q*_calc_) for *n* = 3 electrodes using (a) anodic chronoamperometry, (b) cathodic chronoamperometry, (c) anodic LSV and (d) cathodic LSV. The error bars represent the standard deviation estimated among the three electrodes. Blue dashed lines represent the ideal 1:1 correlation (slope = 1), with the blue shaded regions corresponding to a ± 10% error band from the ideal 1:1 correlation assuming volumetric uncertainty. The solid black lines show the linear fit of the experimental data, framed by a gray shaded region representing a ± 10% variation margin.

All four methodologies exhibited strong linear correlation between *Q*_theo_ and *Q*_calc_; however, differences in slope and dispersion provide valuable insights into individual performance. A-IT-TLC LSV demonstrated excellent linearity (*R* ^2^ = 0.9962) with minimal error bars, confirming high precision and consistency among electrodes. The slope remained closer to unity (1.08) supporting the suitability of this protocol for calibration-free quantification. A-IT-TLC chronoamperometry showed an acceptable correlation (*R* ^2^ = 0.9759) but with slightly higher dispersion and a slope of 0.70, suggesting underestimation of charge and more variability in electrode performance compared to the LSV analogue. In the case of C-IT-TLC, while chronoamperometry displayed a good linear relationship (*R* ^2^ = 0.9898) with lower dispersion than A-IT-TLC, its dynamic range of response was narrower (1–20 μM), and the slope significantly deviates from unity (0.68). On the other hand, greater variability was observed using the cathodic LSV in comparison to the anodic LSV, with the slope being ca. 20% lower than unity.

According to these fundamental outcomes, the systematic deviations observed under both cathodic and anodic chronoamperometric protocols indicate a clear departure from ideal coulometric behavior, thereby limiting the current readiness of these approaches as validated, calibration-free methods. However, rather than being mere experimental artifacts, the investigation of these deviations provides invaluable insights into the mechanistic aspects of the system. Specifically, they shed light on the electrochemical asymmetry that governs the kinetics of ion uptake versus ion release within the thin-layer configuration. Understanding this kinetic mismatch is crucial for refining future theoretical models and optimizing transport dynamics at the interface.

Overall, while the analytical performance depends significantly on the selected protocol, the A-IT-TLC LSV demonstrates the highest fidelity to the calibration-free principle, outperforming the other protocols in terms of precision, reproducibility and agreement with theoretical values when several electrodes are considered in the study. The slope close to unity (1.08) in the correlation with the theoretical charge value along with the low error bars supports its potential as the candidate for a truly calibration-free operation within the strategies herein investigated. This superior performance is associated with the underlying electrochemical mechanism compared to cathodic protocols. Specifically, the sequence prior to the charge readout, involving the ion uptake from the sample (Figure 1c) followed by its depletion (Figure 1d), enables a better control of the interconnected charge-transfer processes.^22^

Although anodic LSV presents more promising fundamental results regarding direct concentration quantification without prior calibration, all four protocols introduced in this work are further evaluated and compared in the subsequent section when analyzing real samples. From a practical perspective, the chronoamperometric approach remains a highly valuable option due to its operational simplicity. Indeed, it is anticipated that the chronoamperometric approach will operate with greater robustness in complex real matrices than LSV. This superior performance is expected because, in principle, pulsed techniques provide a more effective differentiation against background interferences compared to LSV, and furthermore, because the protocols were applied within a concentration range where they exhibit maximum compliance with Faraday’s law. Truly, this work thus establishes a comprehensive comparison between anodic and cathodic readouts in nanomembrane-based amperometric ISEs, filling a prominent gap in current literature.

### Validated Analytical Application in Real Samples

The quantification of the K^+^ content in four different samples (standard KCl solution, human urine, blood serum, and canal water) was accomplished. Both anodic and cathodic protocols were applied to each sample using three equal ITO-POT-membrane electrodes (*n* = 3). The samples were selected to demonstrate the appropriate performance of the sensor in a wide range of complex matrices. As detailed in the Experimental Section, the samples were filtered and diluted with 10 mM NaCl to cover the linear range of responses found for the sensor. This procedure ensured the appropriate conductivity of the more diluted samples while effectively matching the range of response (marked by the ion-exchange capacity of the membrane and confined thin-layer volume). Although this requirement may limit the direct application of the current configuration in decentralized applications, the concept presented herein offers a promising and original alternative to potentiometric ISEs, avoiding intrinsic limitations such as logarithmic signal dependence, temperature sensitivity, and slow membrane equilibration. Importantly, even under dilution, the use of different real matrices supports the applicability of the coulometric readout and demonstrates its potential for reliable ion quantification, providing a solid basis for further extension of the approach to other target analytes.

The K^+^ content in the samples was calculated through five approaches employing: (i) the charge from chronoamperometry readout compared to the calibration curve; (ii) the peak current from LSV compared to the calibration curve; (iii) the peak charge from LSV compared to the calibration curve, (iv) direct calculation via Faraday’s law using the charge from chronoamperometry, and (v) direct calculation via Faraday’s law using the charge from LSV. The samples were also analyzed by IC to evaluate the accuracy of the results. In the cases of K^+^ determination using the Faraday’s law, eq 1 was employed

1 $$Q_{K^{+}}=\mathrm{nF}c_{K^{+}}Ad_{s}$$

where *Q*_K_^+^ is the charge corresponding to the K^+^ transfer, *n* is the moles of electrons transferred per ion transferred (*n* = 1), *F* is the Faraday’s constant (96,485.3 C mol^–1^), *c*_K_^+^ is the concentration of K^+^ in the thin-layer sample, *A* is the area of the thin-layer domain (50.2 ± 0.3 mm^2^) and *d*_s_ is the sample thickness (100 ± 5 μm).

Figure 7 presents the voltammograms, chronoamperograms and corresponding coulometric responses for the four samples using the same ITO-POT-membrane electrode for the anodic (Figure 7a–c) and cathodic methods (Figure 7d–f). In all cases, two well-defined peaks were observed in the voltammograms. In the case of the A-IT-TLC LSV, the first peak appearing between 477 and 484 mV was associated with background ions (mainly Na^+^), and the second peak observed in the range of 740–779 mV corresponded to the K^+^ transfer. For the C-IT-TLC LSV, the first peak appeared between 445 and 455 mV, and the second peak in the range of 643–658 mV, also associated with background ion and K^+^ transfer, respectively. Compared to the anodic mode, the cathodic voltammograms exhibited a smaller potential separation between peaks, resulting in a higher degree of overlap. This overlap may affect the accuracy of charge integration, particularly at higher concentrations where peak broadening becomes even more pronounced. Decreasing the scan rate could serve as an effective strategy to improve peak resolution and ensure more precise integration.

**Figure 7:**
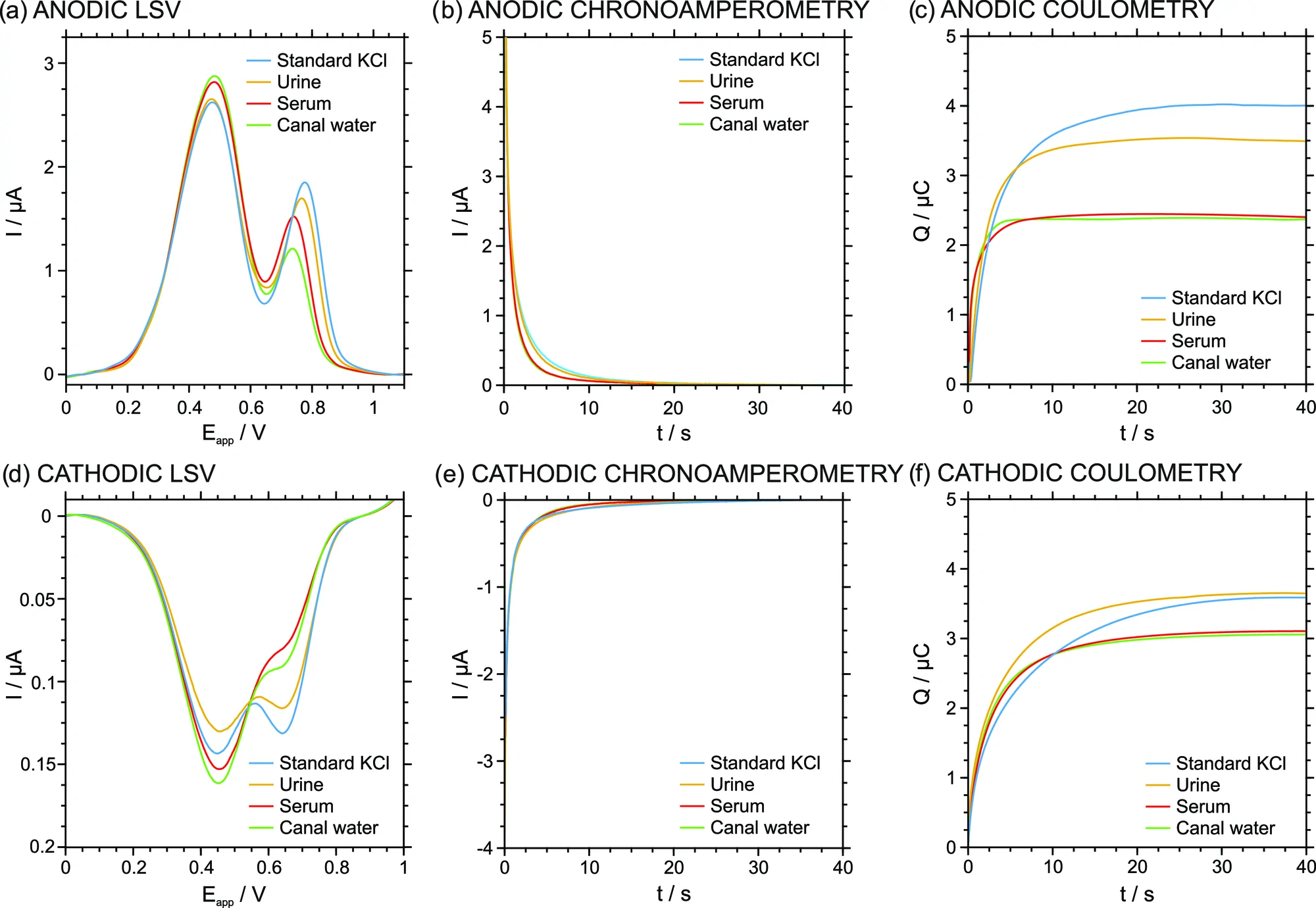
Samples analysis using anodic and cathodic protocols performed by (a, d) LSV; (b, e) chronoamperometry, and (c, f) coulometry.

Table 3 collects the average K^+^ concentration obtained for each sample using the six methodologies explained above. In addition, Figure S11 presents the correlations between the concentrations obtained with each technique and those provided by the IC. Notably, strong linear relationships are observed across all tested protocols. Then, the slopes for all correlations involving the coulometric approach are close to unity, indicating good agreement between the electrochemical measurements and the IC values. In particular, the concentrations calculated via external calibration using the charge from chronoamperometry (Figure S11a) yielded slopes of 0.998 and 1.040 for the A-IT-TLC and C-IT-TLC protocols, respectively. However, when the Faraday’s law was applied to directly calculate the concentration using the charge values from this technique (Figure S11b), the correlation slopes slightly deviated from unity (0.929 for A-IT-TLC and 1.114 for C-IT-TLC). Despite these discrepancies, the relative errors remained below 20%, except for the C-IT-TLC measurement in canal water, highlighting the acceptable accuracy of the coulometric method under the anodic readout. Balancing a precision loss up to a 20% threshold in exchange for a calibration-free operation represents a pragmatic and logical advancement, well-aligned with commercially accepted standards in sensor technology. In clinical settings, for instance, commercial glucose meters routinely operate under regulatory frameworks (e.g., ISO 15197) that tolerate deviations of up to 15–20% in the hands of users. Within this analytical context, sacrificing a strict margin of precision is a logical compromise to bypass the operational burden of frequent calibrations, particularly for decentralized or point-of-care testing.

**Table 3:**
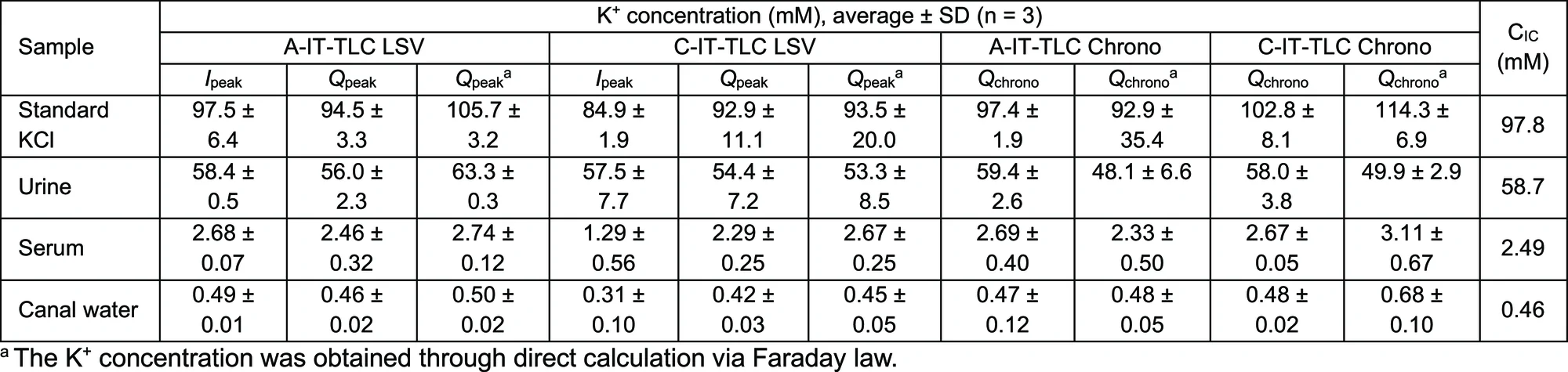
Concentrations of K^+^ Analyzed in Real Samples by Means of Different Methodologies

For concentrations calculated via external calibration using the charge derived from LSV (Figure S11c), the slopes differed moderately from unity (ca. 4% for A-IT-TLC and 5% for C-IT-TLC). When the Faraday’s law was applied using the charge values from *Q*_peak_ (Figure S11d), the resulting slopes improved significantly to 1.000 and 0.999 for A-IT-TLC and C-IT-TC, demonstrating excellent agreement with the IC values. By contrast, when the *I*_peak_ from LSV was used as the analytical signal (Figure S11e), the slope was approximately 10% below unity for the C-IT-TLC protocol, indicating that this parameter offers a less reliable metric for quantification compared to integrated charge-based approaches.

The relative errors of the sample concentrations determined by each method in comparison with IC are summarized in Table S5. It is evident that the accuracy varies significantly depending on the electrochemical protocol and the signal processing method. Most coulometric approaches yielded relative errors below 10%, confirming the good accuracy of the developed protocols. The only exception was the chronoamperometric C-IT-TLC when the concentration was calculated via Faraday’s law, which showed an average error of 20%. Crucially, when external calibration was applied to the same chronoamperometric data, the agreement with IC improved significantly (reducing the average error from 26% to 4%). This indicates that the deviation does not arise from a lack of selectivity or signal reliability. On the other hand, the concentration calculated using Faraday’s law based on the *Q*_peak_ from the C-IT-TLC protocol displayed an average error of 5.6%. Conversely, the use of the cathodic *I*_peak_ as the analytical signal resulted in significantly higher errors (average of 24%). This result indicates once again that peak current is not a suitable parameter for quantitative analysis under the studied conditions.

For a more rigorous statistical evaluation of the agreement between the different methods, Bland-Altman plots were analyzed (Figure S12 in the Supporting Information). Percentage mean biases <15% were found for all the coulometric techniques in comparison with IC measurements. Remarkably, the K^+^ concentration in the standard solution was within the range specified by the provider (100 mM) using all the charge-based methods (<15% of difference). Meanwhile, urine and serum samples revealed a content within physiologically expected levels (2.4–4.8 mM for horse serum^32^ and 20–125 mM for urine^33^). The chronoamperometric methods using external calibration presented a good agreement, with relative mean differences of +2% for A-IT-TLC protocol and +3.6% for C-IT-TC. However, when concentrations were calculated using Faraday’s law, the agreement decreased with a difference of approximately 15% for both methods. Particularly the concentrations calculated with C-IT-TLC protocol exhibited a relative average error with respect to IC of ca. 20%. Regarding the LSV methods, the C-IT-TLC using Faraday’s law is the one that presents higher variability from −20 to +16 mM of difference, but the bias remained low (−2%). Furthermore, the statistical analysis revealed a concentration-dependent proportional bias across the investigated methods. This trend can be attributed to the finite ion-exchange capacity of the membrane at higher concentrations, coupled with residual background contributions that become increasingly prominent at lower concentrations, as noted previously. Overall, the results indicate that, while the coulometric approach enables quantification based on Faraday’s law, its practical accuracy depends on the selected interrogation protocol.

## Conclusions

In this work, we presented a systematic evaluation of a thin-layer microfluidic platform featuring nanomembrane-based ISEs for the calibration-free quantification of cations (specifically, K^+^ as proof of concept), demonstrating that absolute Faradaic accuracy is heavily dictated by the selected electrochemical protocol. By comprehensive comparison of four operational frameworks spanning anodic and cathodic ion-transfer modes, anodic LSV emerged as the protocol capable of strictly validating the ideal calibration-free concept, exhibiting a recovery slope close to unity (1.08) and tight adherence to Faraday’s law within a 1–30 μM range. Conversely, the alternative cathodic and chronoamperometric routes displayed systematic nonideal behavior. When facing real samples, these deviations mitigated, and Bland-Altman statistical analysis confirmed a low bias (<15%) alongside overall good agreement with independent IC measurements across complex matrices such as urine, blood serum, and canal water. Crucially, while direct Faradaic calculation under rapid, pulse-based protocols introduced a localized accuracy trade-off with relative errors reaching a ca. 20% threshold, such a compromise remains highly logical as it aligns with commercially accepted standards, where absolute precision is balanced against the operational simplicity of bypassing frequent calibrations. Ultimately, future implementations will transition toward rigid, microfabricated thin-layer architectures to overcome the volumetric uncertainty inherently associated with compliant spacers, thereby significantly enhancing absolute precision for decentralized sensing.

## Supplementary Material



## Notes

Representative voltammetric responses; detailed procedures for charge calculation from LSV and chronoamperometry; duplicate measurements using LSV, chronoamperometry and coulometry; repeatability and reversibility studies; statistical analysis; analytical results for anodic and cathodic protocols; correlation plots and relative errors in K^+^ concentration determined by different techniques compared to IC (PDF)
