# Supplementary material for: Thin-Layer Sample–Ion-Selective Nanomembrane Architecture for Coulometric Cation Sensing
Source: Anal Chem. 2026 Sep 22;98(39):28689–703. doi: 10.1021/acs.analchem.6c01562 (PMC13647979; doi:10.1021/acs.analchem.6c01562)
Supplement: Supplementary file 1 [file ac6c01562_si_001.pdf]

Supporting Information for:

Thin-Layer Sample–Ion-Selective Nanomembrane Architecture for Coulometric Cation Sensing

Nuria Martínez-Lorca[1], Yujie Liu[2], Gastón A. Crespo[1,2], María Cuartero[1,2,*]

[1]UCAM-SENS, Universidad Católica San Antonio de Murcia, UCAM HiTech, Avda. Andrés Hernández Ros 1, 30107, Murcia, Spain.

[2]Department of Chemistry, KTH Royal Institute of Technology, Teknikringen 30, SE-114 28, Stockholm, Sweden.

*Corresponding author: mariacb@kth.se

Table of Contents

Figures *3*

Figure S1. Representative anodic linear sweep voltammogram of a solution containing Na^+ and K^+ 3

Figure S2. Data treatment for charge calculation from chronoamperometry 3

Figure S3. Data treatment for charge calculation from LSV 4

Figure S4. Example of a cathodic linear sweep voltammogram of a solution containing Na^+ and K^+ 4

Figure S5. Anodic chronoamperogram when $E_{baseline}$ is applied 5

Figure S6. Anodic protocol performed with two identical electrodes 6

Figure S7. Cathodic chronoamperogram when $E_{baseline}$ is applied 7

Figure S8. Cathodic protocol performed with two identical electrodes 8

Figure S9. Repeatability study for anodic and cathodic protocol 9

Figure S10. Reversibility study for anodic and cathodic protocol 10

Figure S11. Correlation between K^+ concentrations determined by different techniques and ion chromatography 11

Figure S12. Bland-Altman plots of the difference of concentrations 12

Tables *13*

Table S1. Repeatability at two concentration levels using anodic and cathodic protocol . 13

Table S2. Reversibility at two concentration levels using anodic and cathodic protocol (sequence lowest → highest concentration) 13

Table S3. Analyticial results using A-IT-TLC protocol 14

Table S4. Analytical results using C-IT-TLC protocol 15

Table S5. Relative errors of K^+ concentration determined by different techniques compared with IC 16

Figures

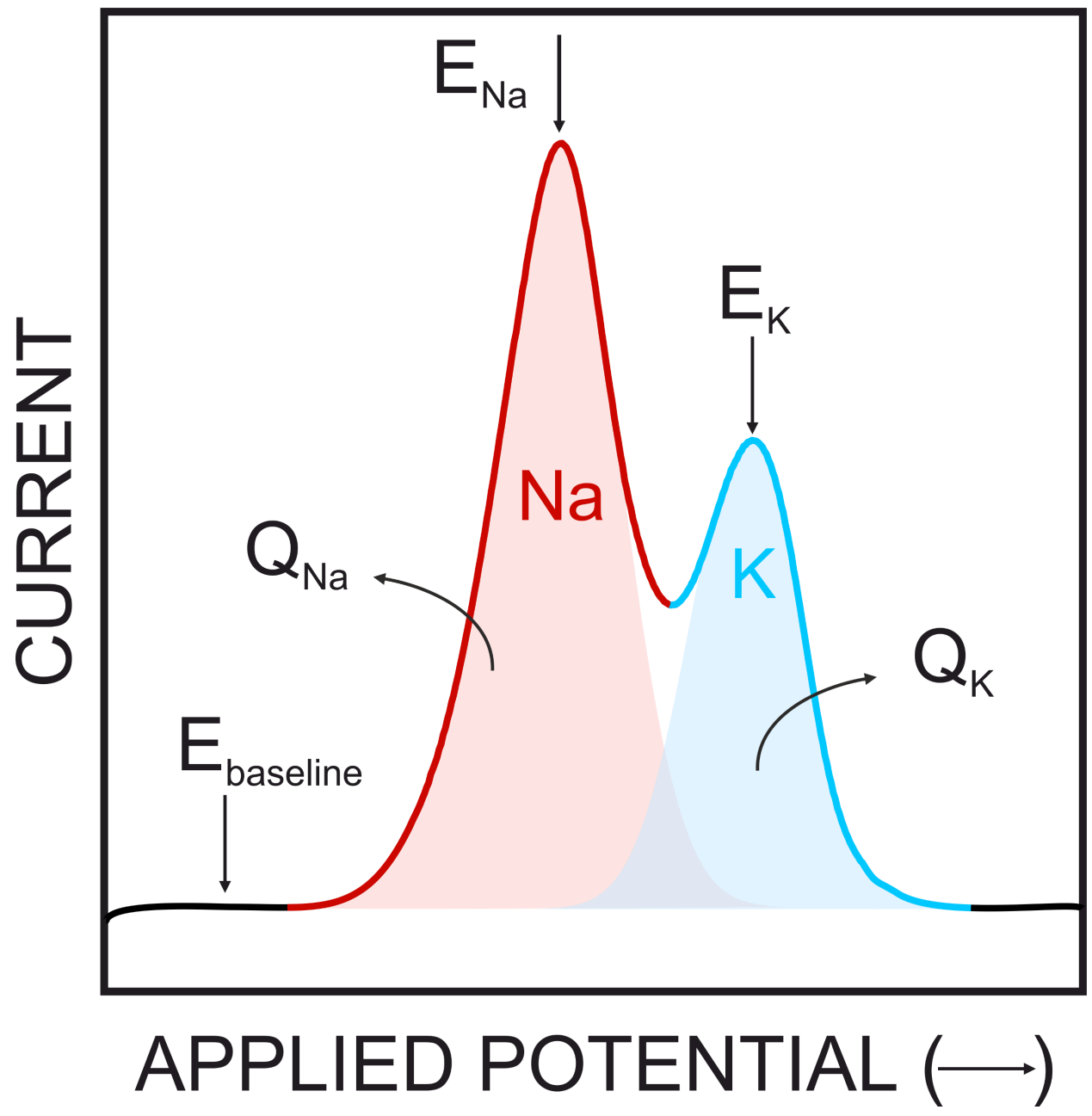

Figure S1. Example of an anodic linear sweep voltammogram of a solution containing Na^{+} and K^{+}.

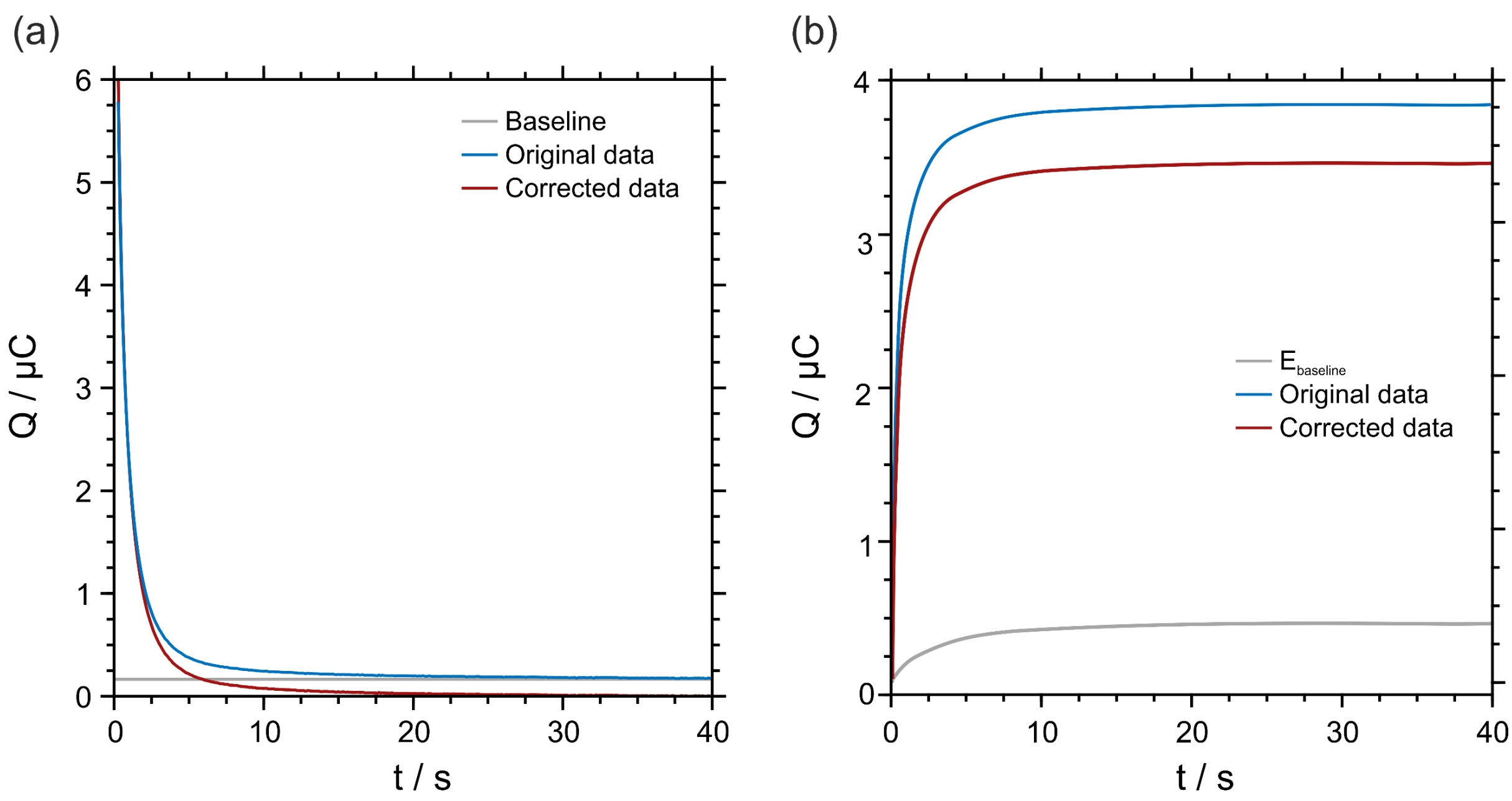

Figure S2. Data treatment for charge calculation from chronoamperometry **(a)** Baseline correction for chronoamperometry. **(b)** Integration and subtraction of the charge associated with non-faradaic processes.

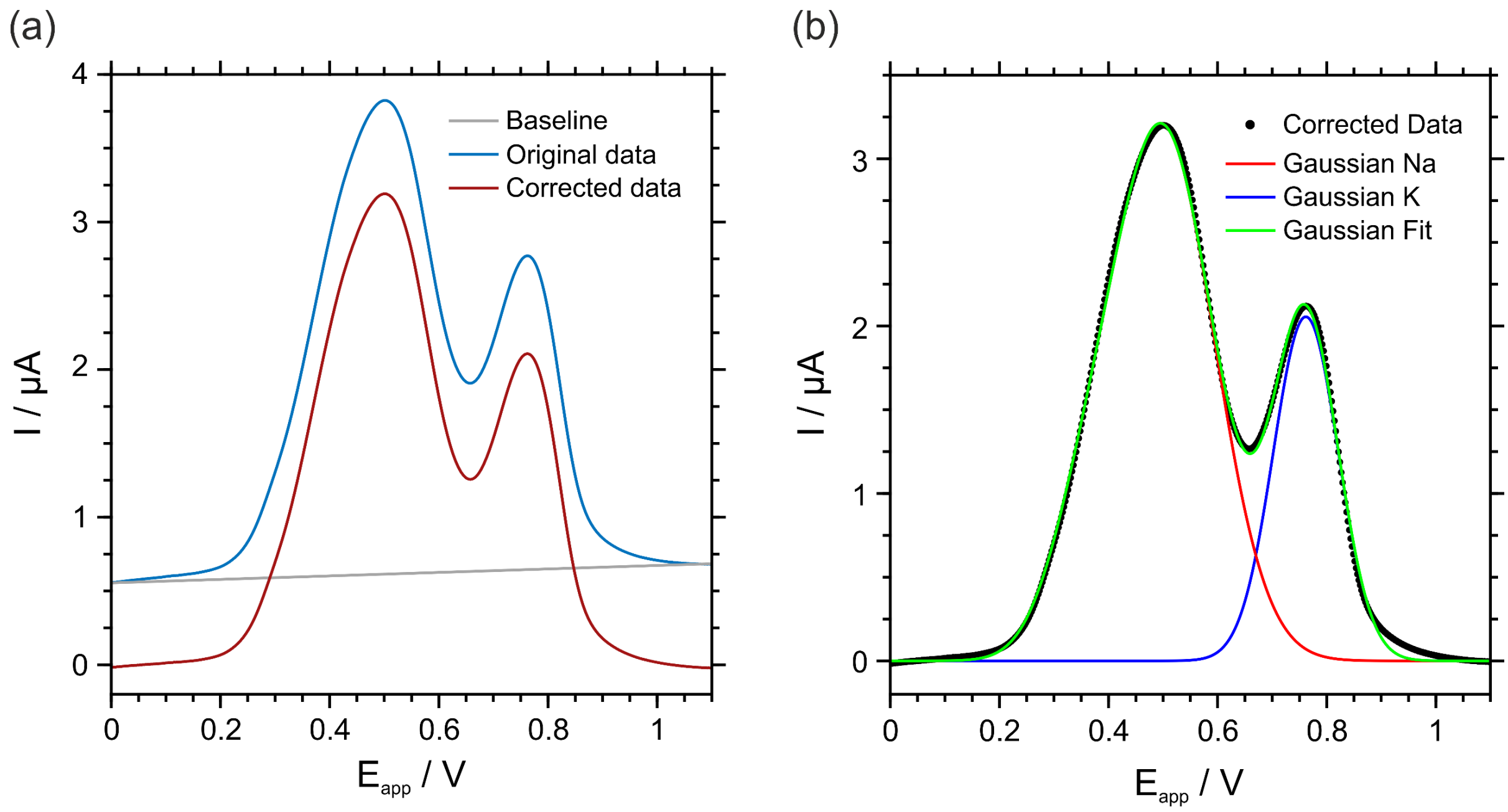

Figure S3. Data treatment for charge calculation from LSV **(a)** Baseline correction for LSV. **(b)** Gaussian fit for the charge calculation of Na^+ and K^+ transfer.

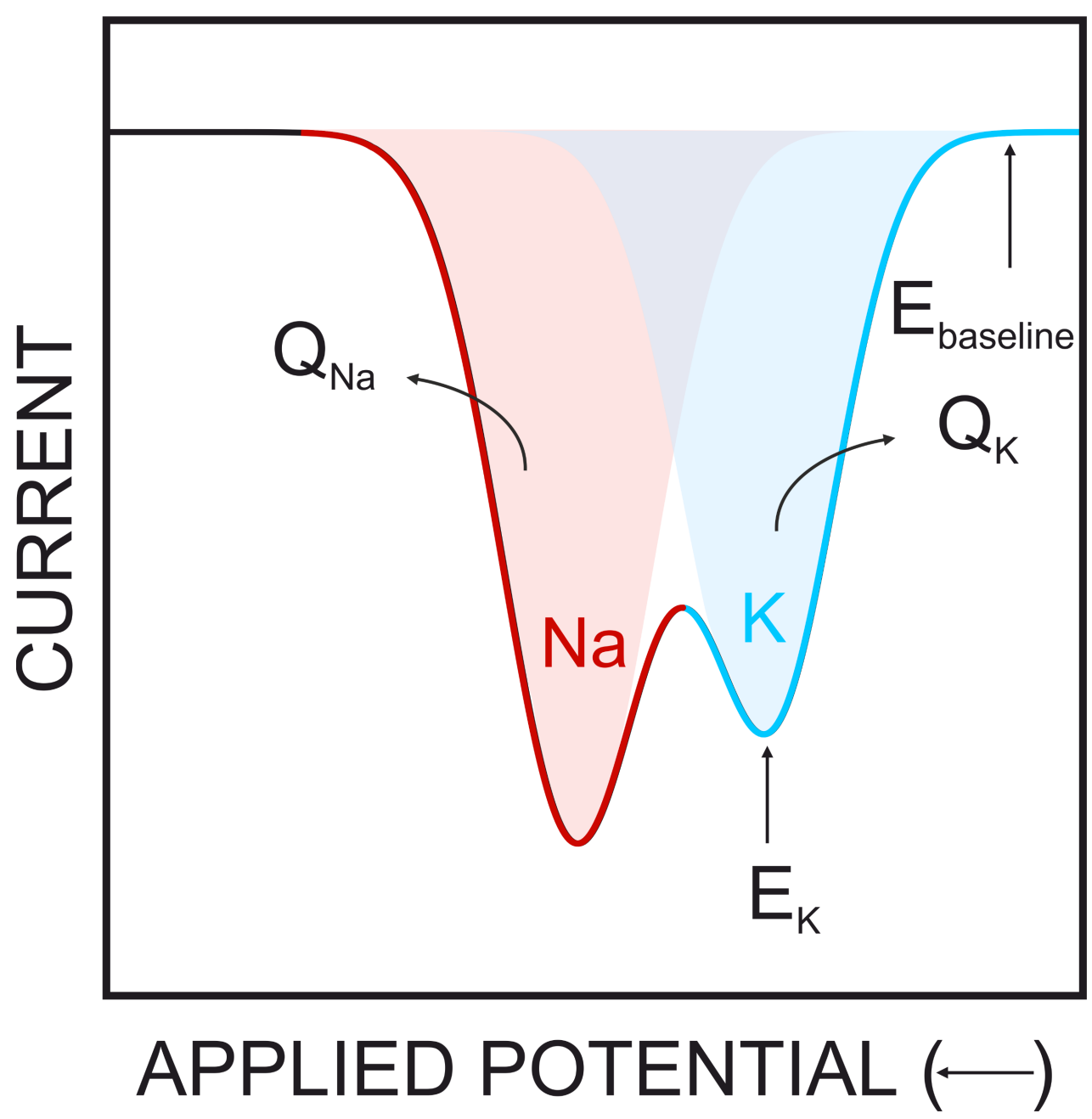

Figure S4. Example of a cathodic linear sweep voltammogram of a solution containing Na^+ and K^+.

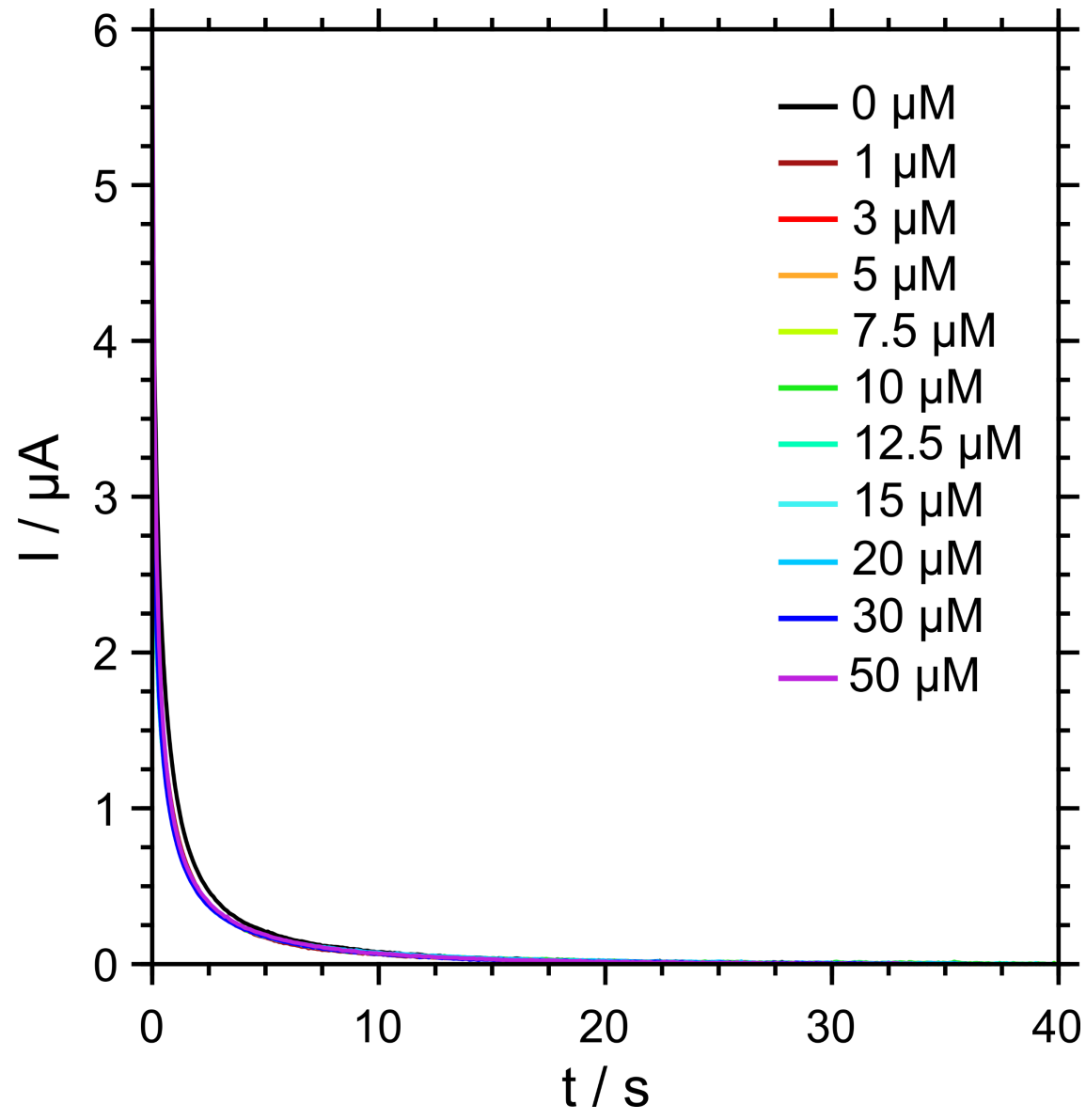

Figure S5. Anodic chronoamperogram when $E_{baseline}$ is applied at increasing KCl concentrations in 10 mM NaCl background at 0.10 V.

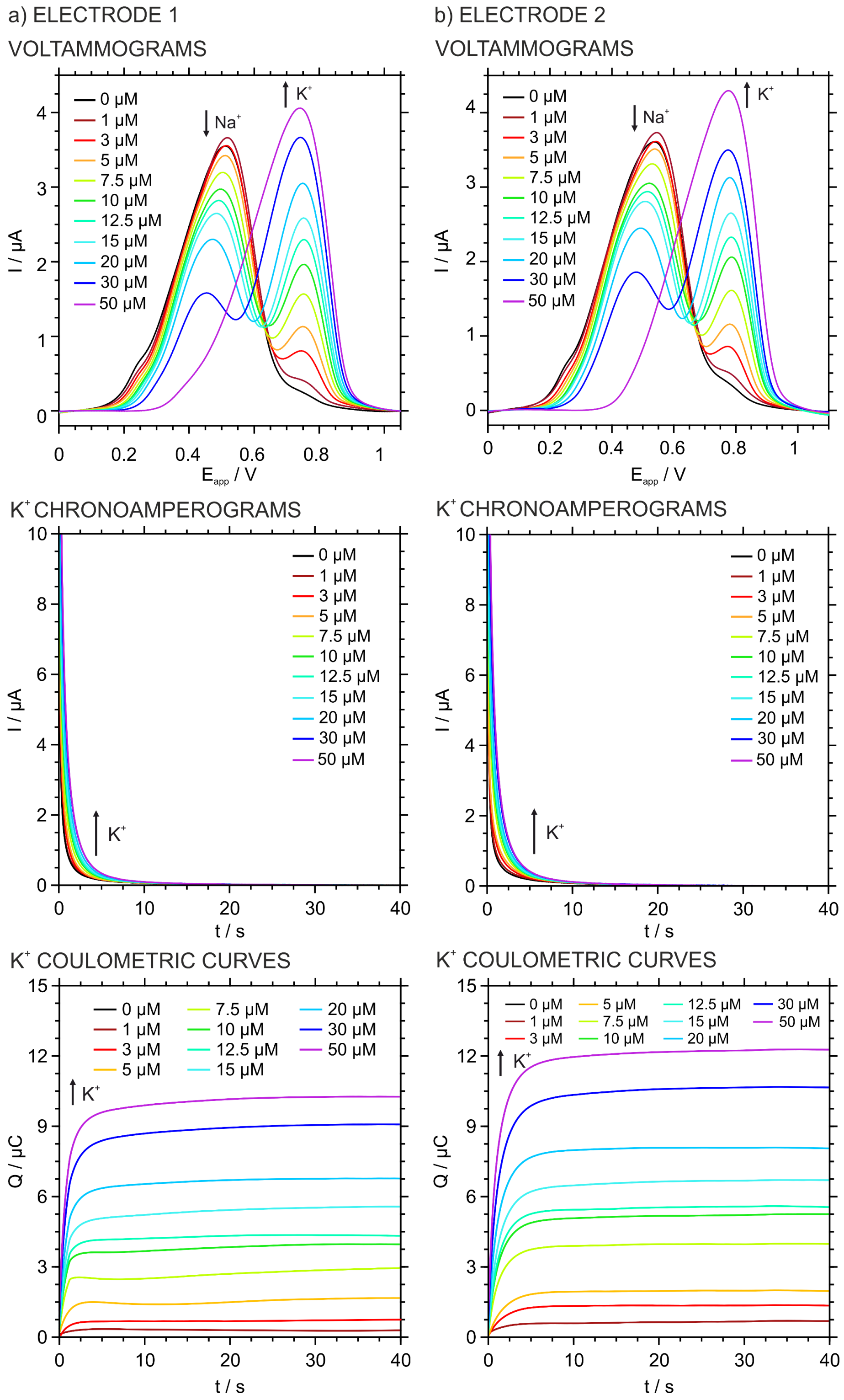

Figure S6. Anodic protocol performed with two identical electrodes **(a)** electrode 1, and **(b)** electrode 2. Electrode 3 is presented in the main manuscript. Electrochemical protocol: $E_{depletion}$ = 1 V during 150 s while pumping at a flow rate of 80 μL min^{-1}, E_{acc} = –0.2 V during 60 s, LSV from –0.2 to 1.1 V, scan rate of 50 mV s^{-1}, : $E_{depletion}$ = 1 V during 150 s while pumping at a flow rate of 80 μL min^{-1}, E_{acc} = –0.2 V during 60 s, $E_{baseline}$ = 0.10 V during 40 s, E_{Na} = 0.50 V during 40 s, E_{K} = 0.80 V during 40 s.

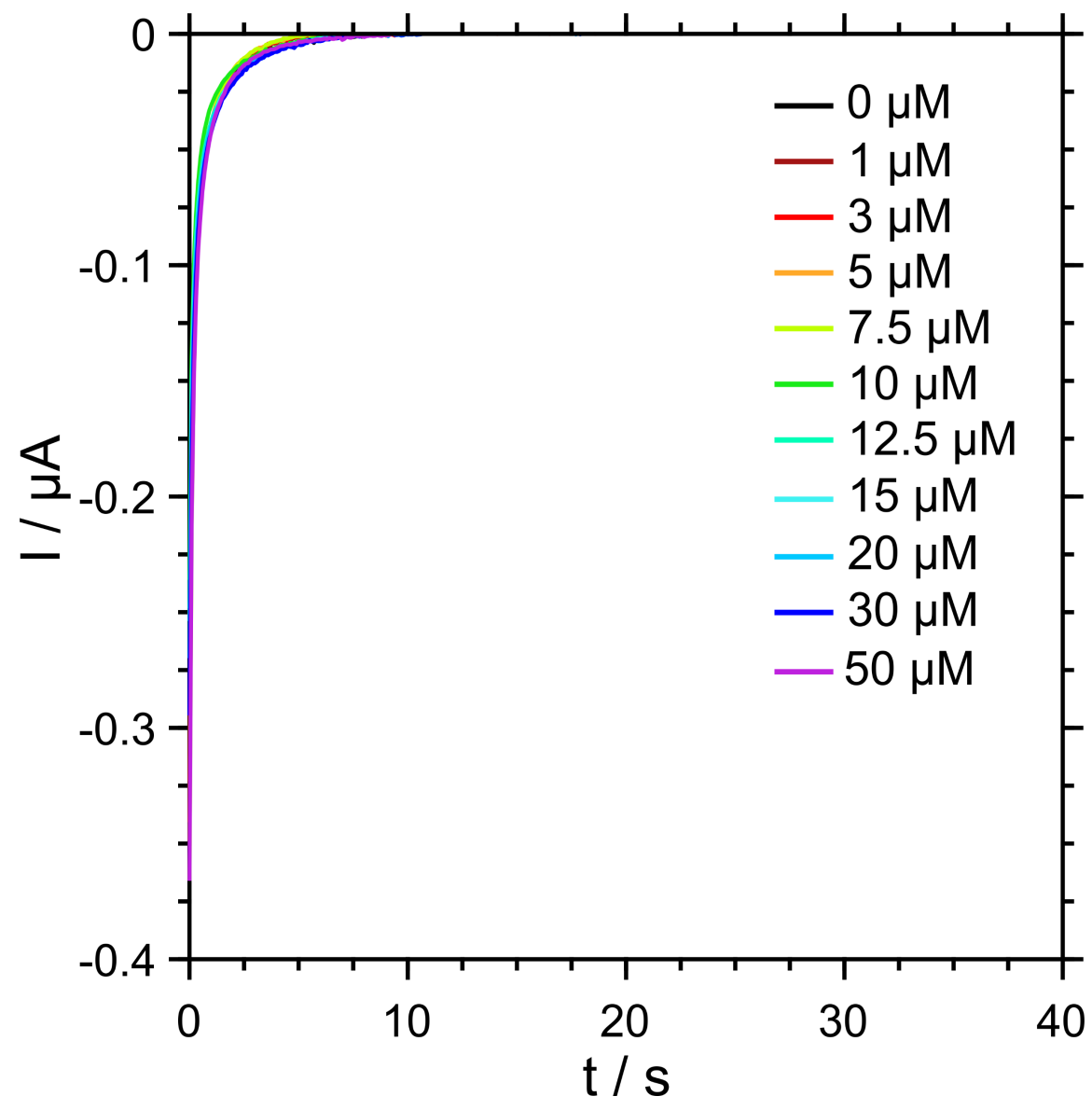

Figure S7. Cathodic chronoamperogram when $E_{baseline}$ is applied at increasing KCl concentrations in 10 mM NaCl background at 0.95 V.

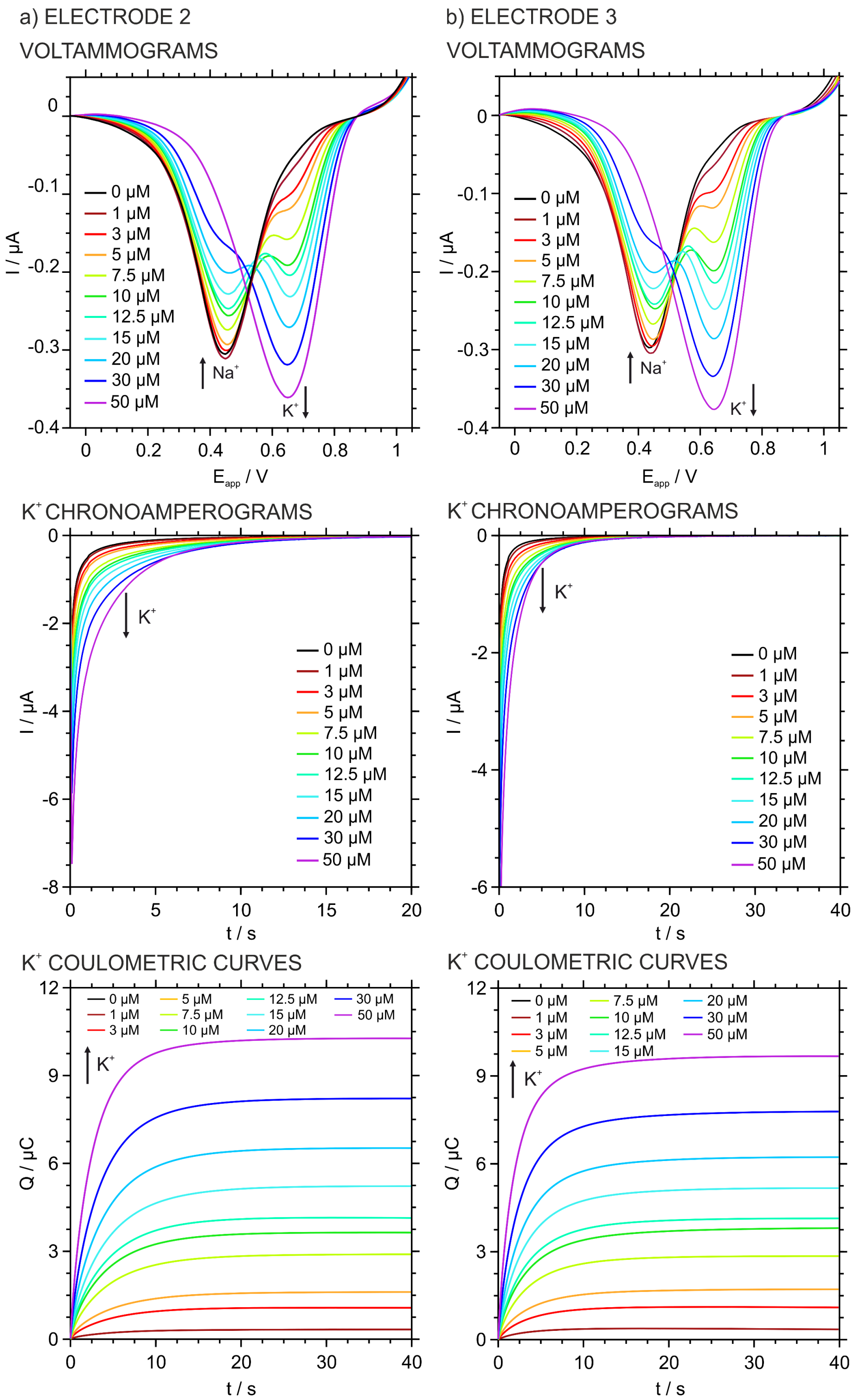

Figure S8. Cathodic protocol performed with two identical electrodes **(a)** electrode 2, and **(b)** electrode 3. Electrode 1 is presented in the main manuscript. Electrochemical protocol: $E_{depletion}$ = 1 V during 150 s while pumping at a flow rate of 80 μL min^{-1}, LSV from 1.1 to –0.1 V, scan rate of 5 mV s^{-1}, $E_{depletion}$ = 1 V during 150 s while pumping at a flow rate of 80 μL min^{-1}, $E_{baseline}$ = 0.95 V during 40 s, E_K = 0.65 V during 40 s.

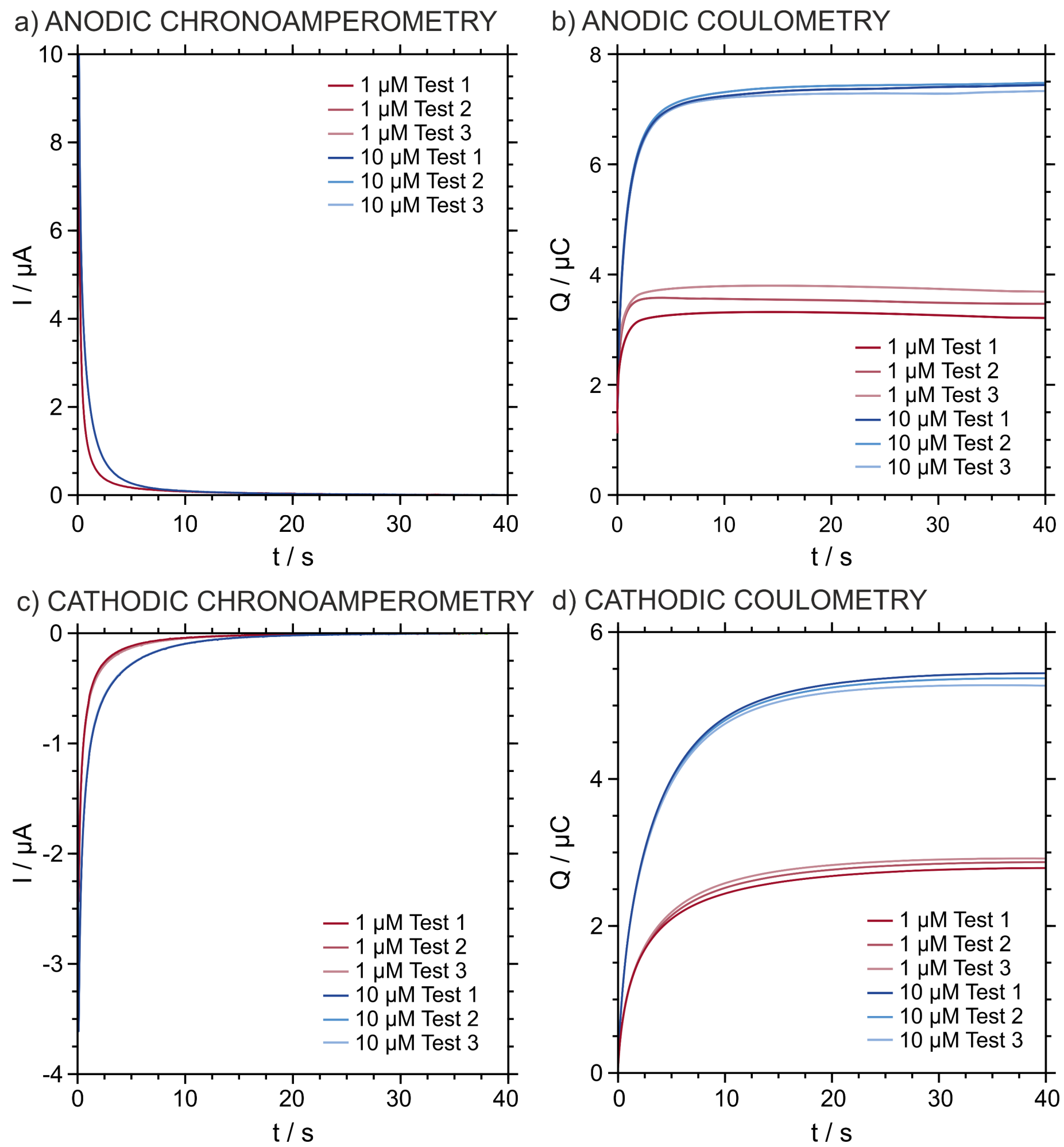

Figure S9. Repeatability study **(a)** Anodic chronoamperograms obtained in triplicate for two K^+ concentrations, 1 and 10 μM in 10 mM NaCl using the same ITO-POT-membrane electrode. **(b)** The corresponding anodic coulometric corrected curves. **(c)** Cathodic chronoamperograms obtained in triplicate for two K^+ concentrations, 1 and 10 μM in 10 mM NaCl using the same ITO-POT-membrane electrode. **(d)** The corresponding cathodic coulometric corrected curves.

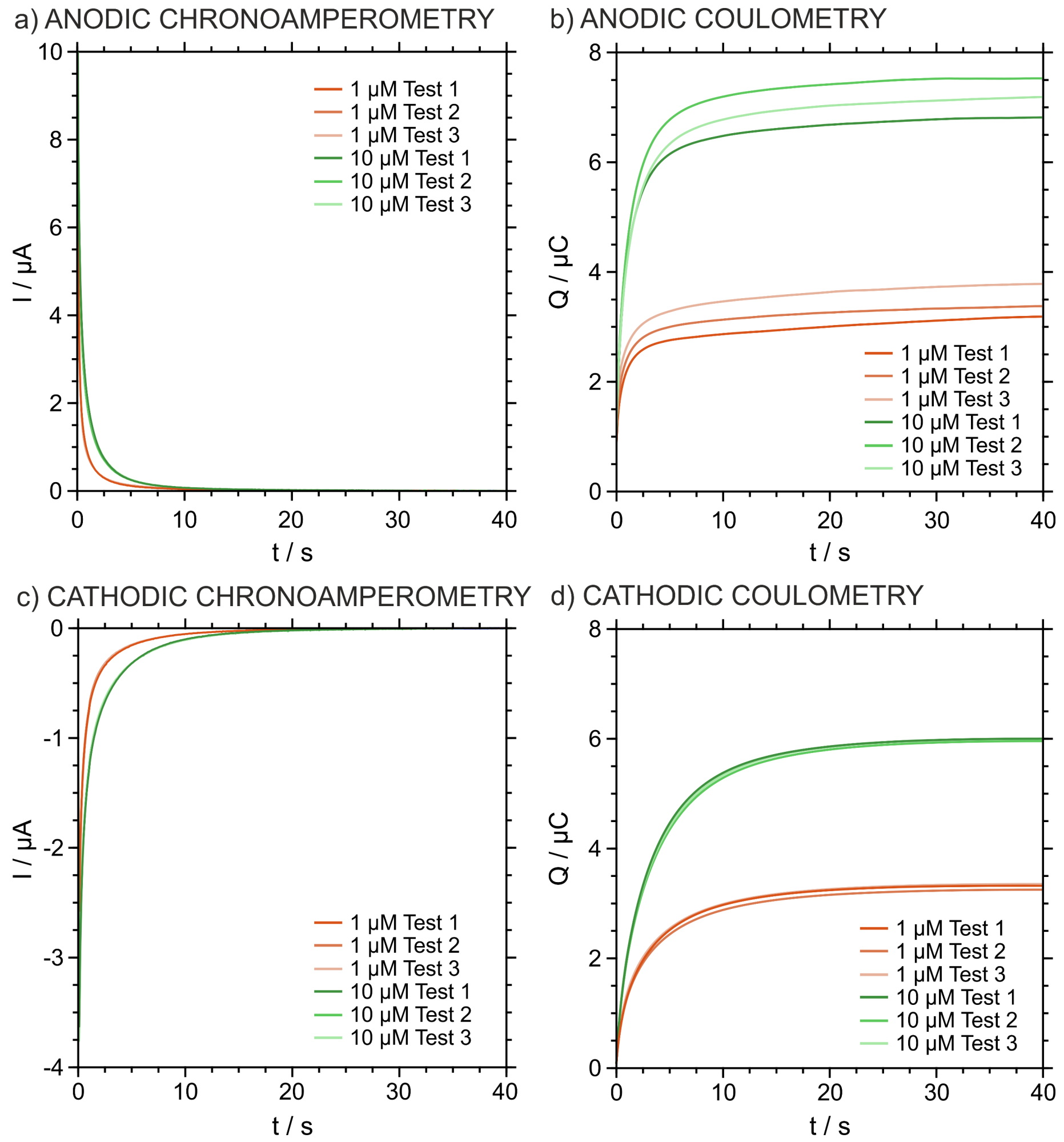

Figure S10. Reversibility study **(a)** Anodic chronoamperograms obtained by alternating two K^+ concentrations, 1 and 10 μM in 10 mM NaCl using the same ITO-POT-membrane electrode. The following sequence was followed: 1 → 10 → 1 → 10 → 1 → 10 μM KCl. **(b)** The corresponding anodic coulometric corrected curves. **(c)** Cathodic chronoamperograms obtained by alternating two K^+ concentrations, 1 and 10 μM in 10 mM NaCl using the same ITO-POT-membrane electrode. The following sequence was followed: 1 → 10 → 1 → 10 → 1 → 10 μM KCl. **(d)** The corresponding cathodic coulometric corrected curves.

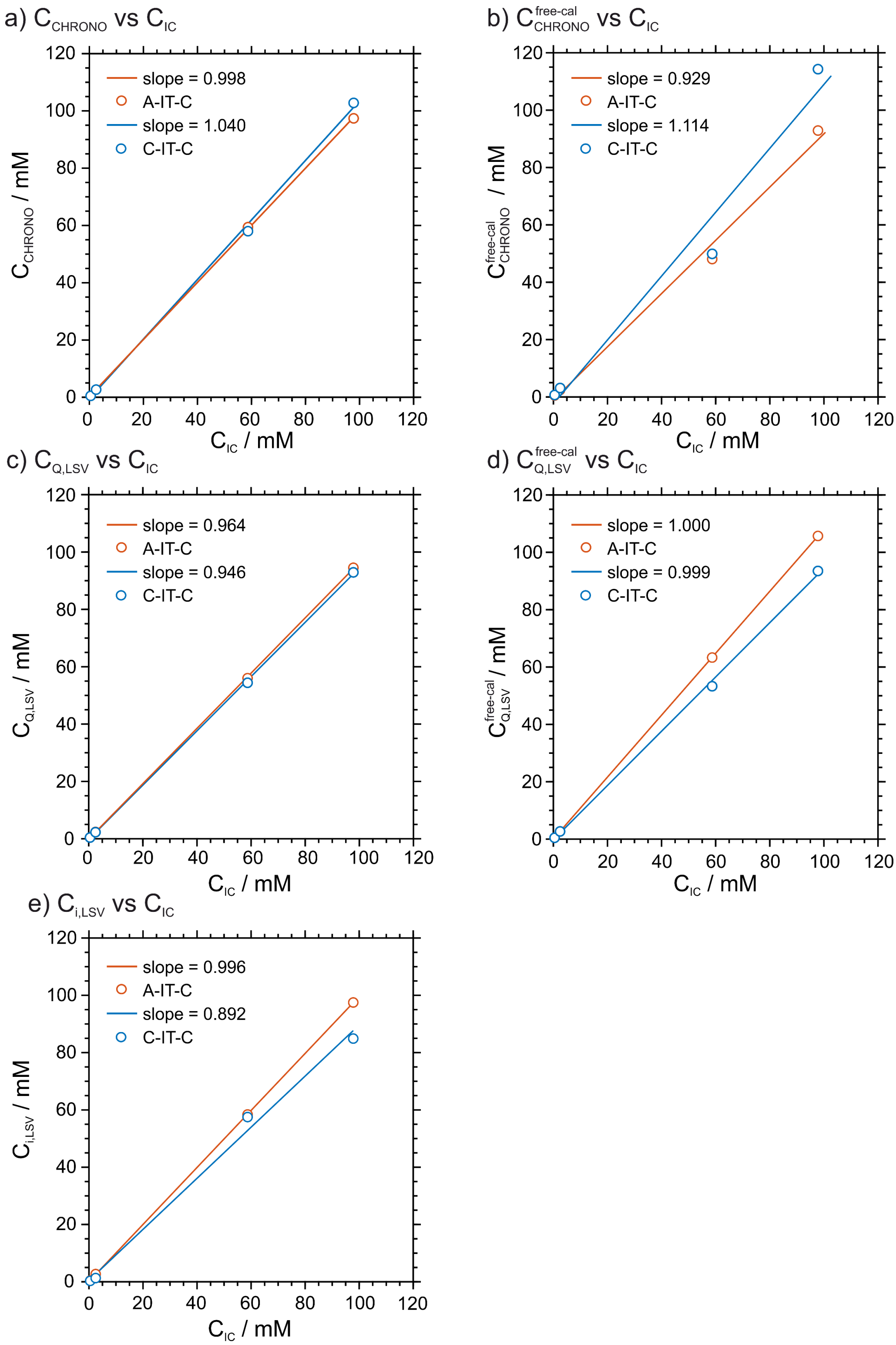

Figure S11. Correlation between K^+ concentrations determined by different techniques and ion chromatography. **(a)** Q_{CHRONO}, **(b)** Faraday law using Q_{CHRONO}, **(c)** Q_{peak} from LSV, **(d)** Faraday law using Q_{peak} from LSV, and **(e)** I_{peak} from LSV.

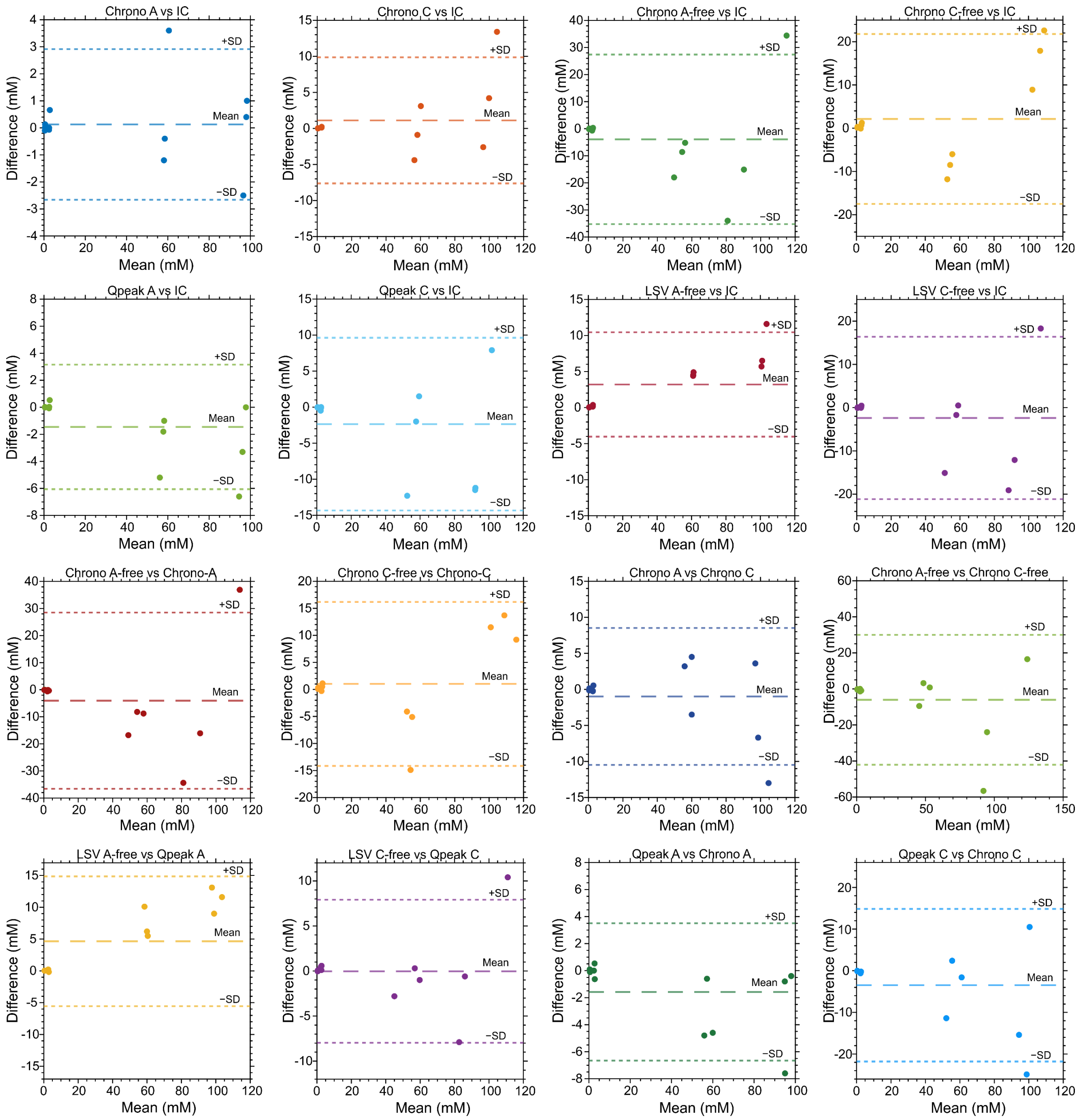

Figure S12. Bland-Altman plots showing differences in concentrations obtained between all the methods.

Tables

Table S1. Repeatability at two concentration levels using anodic and cathodic protocol.

Repeatability	ANODIC CHARGE (μC)		CATHODIC CHARGE (μC)	
	**1 μM**	**10 μM**	**1 μM**	**10 μM**
Measure #1	3.24	7.44	2.79	5.44
Measure #2	3.47	7.48	2.87	5.37
Measure #3	3.69	7.33	2.92	5.27
Average ± SD	3.47 ± 0.18	7.42 ± 0.06	2.86 ± 0.05	5.36 ± 0.07
RSD	5.33	0.85	1.87	1.29

Table S2. Reversibility at two concentration levels using anodic and cathodic protocol following the sequence lowest → highest concentration.

Reversibility	ANODIC CHARGE (μC)		CATHODIC CHARGE (μC)	
	**1 μM**	**10 μM**	**1 μM**	**10 μM**
Measure 1	3.19	6.82	3.32	6.00
Measure 2	3.38	7.53	3.25	5.96
Measure 3	3.78	7.19	3.35	6.00
Average ± SD	3.45 ± 0.30	7.18 ± 0.36	3.31 ± 0.05	5.99 ± 0.02
RSD	8.82	4.95	1.59	0.41

Table S3. Analytical results using A-IT-TLC protocol. **(A)** Coulometric detection of K^+ concentrations in thin-layer samples in a NaCl background solution using chronoamperometry anodic protocol. **(B)** Coulometric detection of K^+ concentrations in thin-layer samples in a NaCl background solution using LSV anodic protocol.

(A)

electrode 1 [a]		electrode 2 [a]		electrode 3 [a]		[b]	[b]		[c]	[d]
Q_K^{chrono} (μC)	c_K^{chrono} (μM)	Q_K^{chrono} (μC)	c_K^{chrono} (μM)	Q_K^{chrono} (μC)	c_K^{chrono} (μM)	Q_K^{chrono} (μC)	c_K^{chrono} (μM)	c_K^{added} (μM)	Efficiency (%)	Relative error (%)
0.55	1.14	0.69	1.42	0.32	0.66	0.52 ± 0.19	1.07 ± 0.39	1.0	107.4	7.4
0.68	1.40	1.35	2.79	1.48	3.06	1.17 ± 0.43	2.42 ± 0.89	3.0	80.5	19.5
1.67	3.45	1.97	4.07	2.06	4.25	1.90 ± 0.20	3.92 ± 0.42	5.0	78.5	21.5
2.95	6.09	3.94	8.13	3.50	7.23	3.46 ± 0.50	7.15 ± 1.02	7.5	95.3	4.7
3.96	8.18	5.25	10.84	4.80	9.91	4.67 ± 0.65	9.64 ± 1.35	10.0	96.4	3.6
4.32	8.92	5.56	11.48	5.42	11.19	5.10 ± 0.68	10.53 ± 1.40	12.5	84.2	15.8
5.57	11.50	6.70	13.83	6.42	13.25	6.23 ± 0.59	12.86 ± 1.21	15.0	85.7	14.3
6.77	13.98	8.06	16.64	8.01	16.54	7.61 ± 0.73	15.72 ± 1.51	20.0	78.6	21.4
9.08	18.75	10.66	22.01	10.66	22.01	10.13 ± 0.91	20.92 ± 1.88	30.0	69.7	30.3
10.26	21.18	12.27	25.33	11.24	23.21	11.26 ± 1.01	23.24 ± 2.08	50.0	46.5	53.5

(B)

electrode 1 [a]		electrode 2 [a]		electrode 3 [a]		[b]	[b]		[c]	[d]
Q_K^{LSV} (μC)	c_K^{LSV} (μM)	Q_K^{LSV} (μC)	c_K^{LSV} (μM)	Q_K^{LSV} (μC)	c_K^{LSV} (μM)	Q_K^{LSV} (μC)	c_K^{LSV} (μM)	c_K^{added} (μM)	Efficiency (%)	Relative error (%)
0.32	0.66	0.27	0.55	0.24	0.50	0.27 ± 0.04	0.58 ± 0.11	1.0	57.6	42.4
1.36	2.81	1.18	2.45	1.59	3.27	1.38 ± 0.20	2.84 ± 0.41	3.0	94.8	5.2
2.41	4.98	2.14	4.42	2.38	4.92	2.31 ± 0.15	4.77 ± 0.31	5.0	95.5	4.5
3.92	8.10	4.00	8.25	3.99	8.24	3.97 ± 0.04	8.20 ± 0.09	7.5	109.3	9.3
5.44	11.23	5.76	11.90	5.67	11.71	5.62 ± 0.17	11.61 ± 0.35	10.0	116.1	16.1
6.81	14.07	6.78	14.00	6.80	14.04	6.80 ± 0.02	14.04 ± 0.04	12.5	112.3	12.3
8.10	16.72	8.14	16.81	8.13	16.78	8.12 ± 0.02	16.77 ± 0.05	15.0	111.8	11.8
10.38	21.43	10.71	22.12	10.79	22.29	10.63 ± 0.22	21.94 ± 0.45	20.0	109.7	9.7
15.26	31.51	14.69	30.33	15.86	32.74	15.27 ± 0.58	31.53 ± 1.20	30.0	105.1	5.1
21.27	43.92	23.36	48.23	20.16	41.63	21.60 ± 1.62	44.59 ± 3.35	50.0	89.2	10.8

[a] The K^+ concentrations were calculated from the chronoamperogram charge via Faraday Law. [b] Average ± SD (n = 3 electrodes). [c] The coulometric efficiency was estimated from the known K^+ concentration added to the sample. [d] The relative error in the calculation of the K^+ concentration was calculated considering that the methodology should present 100% coulometric efficiency.

Table S4. Analytical results using C-IT-TLC protocol. **(A)** Coulometric detection of K^+ concentrations in thin-layer samples in a NaCl background solution using chronoamperometry cathodic protocol. **(B)** Coulometric detection of K^+ concentrations in thin-layer samples in a NaCl background solution using LSV cathodic protocol.

(A)

electrode 1 [a]		electrode 2 [a]		electrode 3 [a]		[b]	[b]		[c]	[d]
Q_K^{chrono} (μC)	c_K^{chrono} (μM)	Q_K^{chrono} (μC)	c_K^{chrono} (μM)	Q_K^{chrono} (μC)	c_K^{chrono} (μM)	Q_K^{chrono} (μC)	c_K^{chrono} (μM)	c_K^{added} (μM)	Efficiency (%)	Relative error (%)
0.48	0.99	0.33	0.68	0.35	0.72	0.39 ± 0.08	0.80 ± 0.17	1.0	80.0	20.0
1.06	2.19	1.07	2.21	1.10	2.27	1.08 ± 0.02	2.22 ± 0.04	3.0	74.4	25.6
1.85	3.82	1.61	3.32	1.72	3.55	1.73 ± 0.12	3.56 ± 0.25	5.0	71.6	28.4
2.87	5.93	2.90	5.99	2.85	5.88	2.87 ± 0.03	5.93 ± 0.05	7.5	79.4	20.6
4.08	8.42	3.64	7.52	3.81	7.87	3.84 ± 0.22	7.93 ± 0.46	10.0	79.7	20.3
4.62	9.54	4.14	8.55	4.14	8.55	4.30 ± 0.28	8.88 ± 0.57	12.5	71.3	28.7
5.60	11.56	5.22	10.78	5.17	10.67	5.33 ± 0.24	11.00 ± 0.49	15.0	73.7	26.3
6.78	14.00	6.52	13.46	6.23	12.86	6.51 ± 0.28	13.44 ± 0.57	20.0	67.5	32.5
8.77	18.11	8.22	16.97	7.78	16.06	8.26 ± 0.50	17.05 ± 1.02	30.0	57.1	42.9
9.11	18.81	10.27	21.20	9.67	19.96	9.68 ± 0.58	19.99 ± 1.20	50.0	40.1	59.9

(B)

electrode 1 [a]		electrode 2 [a]		electrode 3 [a]		[b]	[b]		[c]	[d]
Q_K^{LSV} (μC)	c_K^{LSV} (μM)	Q_K^{LSV} (μC)	c_K^{LSV} (μM)	Q_K^{LSV} (μC)	c_K^{LSV} (μM)	Q_K^{LSV} (μC)	c_K^{LSV} (μM)	c_K^{added} (μM)	Efficiency (%)	Relative error (%)
0.45	0.93	0.34	0.70	0.16	0.33	0.32 ± 0.15	0.63 ± 0.42	1.0	63.0	37.0
1.19	2.46	0.82	1.69	0.84	1.73	0.95 ± 0.21	1.96 ± 0.43	3.0	65.4	34.6
1.69	3.49	1.43	2.95	1.66	3.43	1.59 ± 0.14	3.29 ± 0.29	5.0	65.8	34.2
2.98	6.15	2.89	5.97	2.25	4.65	2.71 ± 0.40	5.59 ± 0.82	7.5	74.5	25.5
3.90	8.05	2.97	6.13	3.22	6.65	3.36 ± 0.48	6.94 ± 0.99	10.0	69.4	30.6
4.70	9.70	4.30	8.88	4.39	9.06	4.46 ± 0.21	9.21 ± 0.43	12.5	73.7	26.3
6.07	12.53	5.44	11.23	6.56	13.54	6.02 ± 0.56	12.44 ± 1.16	15.0	82.9	17.1
8.03	16.58	8.15	16.83	8.45	17.45	8.21 ± 0.22	16.95 ± 0.45	20.0	84.8	15.2
11.53	23.80	10.31	21.29	10.87	22.44	10.90 ± 0.61	22.51 ± 1.26	30.0	75.0	25.0
14.60	30.14	13.12	27.09	13.81	28.51	13.84 ± 0.74	28.58 ± 1.53	50.0	57.2	42.8

[a] The K^+ concentrations were calculated from the chronoamperogram charge through Faraday Law. [b] Average ± SD (n = 3 electrodes). [c] The coulometric efficiency was estimated from the known K^+ concentration added to the sample. [d] The relative error in the calculation of the K^+ concentration was calculated considering that the methodology should present 100% coulometric efficiency.

Table S5. Relative errors of K^+ concentration determined by different techniques compared with ion chromatography.

Sample	Relative error in K^+ concentration vs IC (%)									
	LSV A-IT-C			LSV C-IT-C			Chrono A-IT-C		Chrono C-IT-C	
	I_{peak}	Q_{peak}	Q_{peak}*	I_{peak}	Q_{peak}	Q_{peak}*	Q_{chrono}	Q_{chrono}*	Q_{chrono}	Q_{chrono}*
Standard KCl	0.3	3.4	8.1	13.2	5.4	4.4	0.4	5.0	5.1	16.8
Urine	0.5	4.6	7.8	2.1	7.3	9.3	1.1	18.1	1.3	14.9
Serum	7.6	1.2	9.9	48.1	8.1	7.2	7.9	6.4	7.1	24.9
Canal water	6.5	0.5	8.7	32.2	8.2	1.5	2.9	5.4	4.2	48.7

*Concentration calculated using Faraday's law
